# Lipidomic study of the influence of dietary fatty acids on structural lipids of cold-water nudibranch molluscs

**DOI:** 10.1038/s41598-019-56746-8

**Published:** 2019-12-27

**Authors:** Andrey B. Imbs, Valeria P. Grigorchuk

**Affiliations:** 1 National Scientific Center of Marine Biology, Far Eastern Branch, Russian Academy of Sciences, 17 Palchevskogo str., 690041 Vladivostok, Russian Federation; 2 Federal Scientific Center of the East Asia Terrestrial Biodiversity (Institute of Biology and Soil Science), Far Eastern Branch of the Russian Academy of Sciences, 159 Stoletija Str., 690022 Vladivostok, Russian Federation

**Keywords:** Lipidomics, Lipids, Boreal ecology, Ecosystem ecology

## Abstract

Nudibranch molluscs occur in marine ecosystems worldwide and prey on numerous invertebrate species. During feeding, dietary fatty acids (FAs) unusual for nudibranchs are transferred to their lipids. Normal biomembrane functions require stable composition of structural polar lipids (PL), but the pathways of dietary FA utilization to PL in nudibranchs still remain unknown. A combination of chromatography and tandem high-resolution mass spectrometry was used to determine total lipid, PL, FA, and PL molecular species composition of two cold-water species of *Dendronotus*, which then were compared with those of *Tritonia tetraquetra*. The use of FA trophic markers showed that *Dendronotus* sp. and *T. tetraquetra* prey on different soft corals, while *D. robustus* may consumes hydrocorals and bryozoans. Nudibranch FA profiles were strongly modified by dietary FAs but their PL profilers were similar. Dietary FAs are not included in ceramide aminoethylphosphonate and inositol glycerophospholipids, but directed to ethanolamine, choline, and serine glycerophospholipids and, in some cases, form isobaric molecular species with different FA chain lengths. For such isobaric species, nudibranchs reduce the length of alkyl groups when very-long-chain FAs are obtained with diet. This molecular mechanism may explain the adaptation of nudibranch membrane structure to dietary input of unusual FAs.

## Introduction

Nudibranchs are a large group of marine molluscs (Mollusca: Gastropoda: Nudibranchia)^1^, sometimes casually called sea slugs, which includes almost 3000 species inhabiting marine benthic ecosystems worldwide from Arctic to Antarctica. Nudibranchs are an important chain in marine food webs. Numerous studies of nudibranch feeding ecology showed that their diet includes soft corals, reef-building corals, sponges, bryozoans, tunicates, barnacles, sea anemones, jellyfish, ophiuroids, colonial hydroids, and other nudibranchs^2–5^. Nudibranchs are carnivorous, but detritus and plankton may comprise some part of their diet^6^. Many nudibranch species exhibit high dietary specialization; some of them contain symbiotic bacteria^7^ or dinoflagellates captured with prey^6,8^. Identification of food sources for nudibranchs is important for understanding their ecology and description of trophic interactions in marine benthic ecosystems. The method of biochemical tracers, such as lipid and fatty acid (FA) analysis, provides essential information on marine food webs^9^. FAs have been used as markers to trace predator-prey relationships in the world’s ecosystems for more than 40 years^10,11^.

Each taxonomic group of nudibranchs’ preys is characterized by specific low-molecular weight compounds, which are consumed by nudibranchs and can be recognized in their tissues^12,13^. Lipids are the most important part of the low-molecular weight compounds in the diet of consumers and cover a considerable part of its energy demands. Dietary lipids are decomposed in consumer’s organism to acylglycerols and FAs, which can be used for biosynthesis of new acyl lipids^14^. Specific dietary FAs in total lipids of nudibranchs have been earlier analysed for identifying of the food sources of these molluscs. Rare very-long-chain FAs synthesized by marine sponges were detected in FAs of total lipids of several nudibranch species indicating sponges as possible items of their food^15^. Unusual tetracosapolyenoic acids (TPA) synthesized by octocorals were used as dietary tracers to confirm the predator–prey relationship between the cold-water nudibranch *Tritonia tetraquetra* and the soft coral *Gersemia fruticosa*^16^, as well as between the temperate-water nudibranch *Armina maculata* and the sea pen *Veretillum cynomorium*^17^.

Intercellular FA biosynthesis and dietary lipids are known to be the main origins of total FAs in marine animals. Dietary lipids were found to have a considerable influence on total FA profile in nudibranchs^15–19^. The differences observed in the FA composition of egg masses of the *Polycera* nudibranchs preying on the bryozoon *Bugula neritina* and *Berghia* nudibranchs preying on the sea anemone *Sagartia troglodites* were attributed to the differences in the FA composition of their diets^18^. It was shown that the different FA composition of symbiotic and aposymbitic forms of the anemone *Aiptasia pallida* reflected in the FA compositions of egg masses of the tropical nudibranch *Aeolidiella stephanieae* that feeds on these anemone forms^19^. According to earlier studies, total FAs of nudibranchs may contain more than 20% of specific dietary FAs that cannot be synthesized in nudibranch tissues^15–17^. The symbiotic partnerships with bacteria also represent an important source of FAs in nudibranchs^7^.

Dietary FAs can be utilized for building of neutral lipids, which serve as an energy storage, and polar lipids composing the structural base of biological membranes. Controlling the utilization of dietary FAs for the biosynthesis of new polar lipid molecules is very important, because the FA composition of these molecules substantially determine many properties of cell membranes^20^. Nevertheless, an intensive “flux” of unusual dietary lipids and FAs seems to strongly modify the profile of polar lipid molecular species (polar lipidome) of nudibranchs with a specialized type of feeding (stenophagy)^15,21^. The first analysis of the polar lipidome of the nudibranch *T. tetraquetra* showed that unusual TPA, concentrated in serine glycerophospholipids (PS) of its prey^22,23^, are redistributed in the consumer to other polar lipid classes and accumulated in all tissues^21^. Does it mean that nudibranchs are what they eat?

In this study, total lipid and FA compositions of two cold-water nudibranchs, *Dendronotus robustus* and *Dendronotus* sp., were determine for the first time and compared with that of the cold-water nudibranch *T. tetraquetra* collected in the same location (Kuril Islands, the Sea of Okhotsk). Dietary preferences of these three species were studied using the method of lipid and FA trophic markers. Chemical structure and composition of all molecular species of polar lipids (polar lipidome) of the *Dendronotus* species were analysed by tandem high-resolution mass spectrometry for the first time and compared with those of *T. tetraquetra*^21^. The influence of diet on the structural lipid composition of the nudibranchs and the possible molecular mechanisms of adaptation of their polar lipidomes to dietary input are discussed.

## Results

### Lipid class and fatty acid composition

To characterize total lipids (TL) of the animals studied, the lipid class composition was analysed by thin-layer chromatography (TLC) (Supplementary Fig. S1). Polar lipids (PL), sterols (ST), free fatty acids (FFAs), triacylglycerols (TG), sterol esters (SE), and wax esters (WE) were quantified in TL extracts from two nudibranch species of the genus *Dendronotus* (Supplementary Table S1). TL constituted more than 55% of PL. One phosponolipid, ceramide aminoethylphosphonate (CAEP), and several glycerophospholipids (GPL), such as ethanolamine glycerophospholipids (PE), choline glycerophospholipids (PC), serine glycerophospholipids (PS), and inositol glycerophospholipids (PI), were identified in PL of the *Dendronotus* (Supplementary Table S2). The major PL classes were PE and PC.

To identify the FA markers of food sources, that may be consumed by the nudibranchs and, hence, indicate these sources, the FA composition of animal total lipids was analysed. Gas chromatography–mass spectrometry (GC–MS) allowed the identification of 36 compounds in total FAs obtained by hydrolysis of nudibranch TL (Supplementary Table S3, Supplementary Figs. S2–S4). Several mollusc-specific non-methylene-interrupted (NMI) FAs (20:2 NMI and 22:2 NMI) were found. A group of saturated (16:0, 18:0), monounsaturated (18:1n-9, 20:1n-9), and polyunsaturated (20:4n-6, 20:5n-3) fatty acids comprised more than a half of total FAs in both species. A sharp difference in the percentage of docosahexaenoic acid (22:6n-3) and tetracosapolyenoic acids (TPA, 24:5n-6 and 24:6n-3) was observed between the two *Dendronotus* species (Supplementary Table S3). Total FAs of *D. robustus* contained 12.1% of 22:6n-3 and 1.4% of TPA, while total FAs of *Dendronotus* sp. contained 0.7% of 22:6n-3 and 24.8% of TPA.

**Figure 1:**
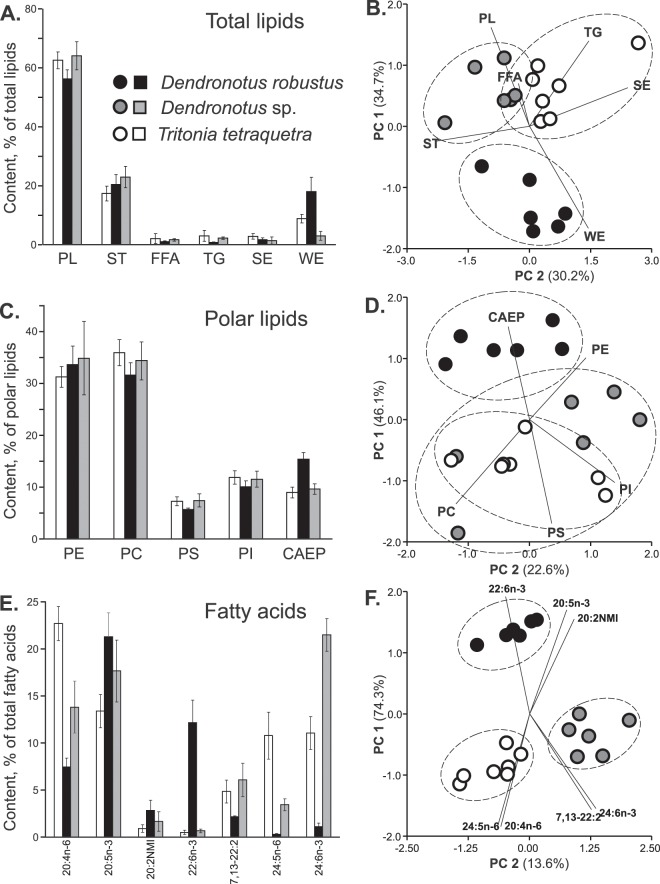
Distribution of lipids and fatty acids. (**A**) Total lipid composition (mean ± SD, see full names in the text) and (**B**) the result of principal components analysis (PCA). (**C**) Polar lipid composition and (**D**) the result of PCA. (**E**) Composition of the principal fatty acids and (**F**) the result of PCA. Ellipses in (**B**, **D**, and **F**) are drawn only to indicate the grouping of the different nudibranch species relative to each other. Three nudibranch molluscs were compared: *Tritonia tetraquetra* (*n* = 7, white bars and circles), *Dendronotus robustus* (*n* = 6, black bars and circles), and *Dendronotus* sp. (*n* = 6, grey bars and circles). Full data on lipid and fatty acid composition and significant differences (one-way ANOVA) in their concentration between three species are provided in Supplementary Tables S1–S3.

A comparison of both TL and PL compositions of *D. robustus*, *Dendronotus* sp., and *T. tetraquetra*, described earlier^16,21^, was performed by applying a one-way analysis of variance (ANOVA) and a principal component analysis (PCA). All TL or PL classes were used as variables for PCA. Similar comparisons between the three nudibranch species were made for their FA compositions. PCA was performed on seven FA variables (20:4n-6, 20:5n-3, 20:2 NMI, 22:6n-3, 7,13–22:2, 24:5n-6, and 24:6n-3). The ANOVA results are summarized in Supplementary Tables S1–S3. The outcome of PCA is visualized in Fig. 1. Despite considerable overlap between *Dendronotus* sp. and *T. tetraquetra*, specimens of *D. robustus* can be recognized in the TL and PL score plots of the first two principal components (Fig. 1B,D). The higher levels of WE and CAEP (F_2,16_ = 37.47, p < 0.0001 and F_2,16_ = 61.30, p < 0.0001, respectively) distinguished *D. robustus* from specimens of *Dendronotus* sp. and *T. tetraquetra* (Fig. 1A,C) All nudibranch species were clearly separated by their FA composition (Fig. 1F). In FA score plot (Fig. 1F), *Dendronotus* sp. is located closer to *T. tetraquetra* than to *D. robustus*, thus indicating a similarity between the FA profiles of *Dendronotus* sp. and *T. tetraquetra*, belonging to different genera. The high content of both 24:5n-6 (10.5%) and 24:6n-3 (10.7%) distinguished *T. tetraquetra* from *Dendronotus* sp., in which 24:6n-3 (21.4%) dominates TPA (Fig. 1E). The percentage of TPA in total FAs of *D. robustus* (1.4%) was 15-fold lower than that of other nudibranch species. (Fig. 1E, Supplementary Table S3). At the same time, *D. robustus* contained significantly higher (F_2,16_ = 166.59, p < 0.0001) concentration of branched FAs (i-16:0, br-17:1, 7-Me-16:1n-10, i-17:0, ai-17:0, 17:0, br-18:1, i-18:0) than *Dendronotus* sp. and *T. tetraquetra* (Supplementary Table S3).

### Molecular species of polar lipids

Polar lipidome of nudibranchs was analysed in order to assess the influence of dietary FAs on the profile of structural lipids. Chemical structure and content of PL molecular species of two *Dendronotus* species were determined by high-performance liquid chromatography (HPLC) with tandem high-resolution mass spectrometry and compared with those of *T. tetraquetra* described earlier^21^. A total of 91 molecular species of PL were identified (Supplementary Table S4, Supplementary Fig. S5–S9). (CAEP molecular species are abbreviated as Xb/Y, where X is sphingoid base, and Y is acyl group. Alkylacyl GPL molecular species are abbreviated as Xe/Y, where X is alkyl group, and Y is acyl group. Diacyl GPL molecular species are abbreviated as X/Y, where X and Y acyl groups are presumably in *sn*-1 and *sn*-2 positions, respectively. Direct identification of the *sn*-1 and *sn*-2 positions was not performed).

**Figure 2:**
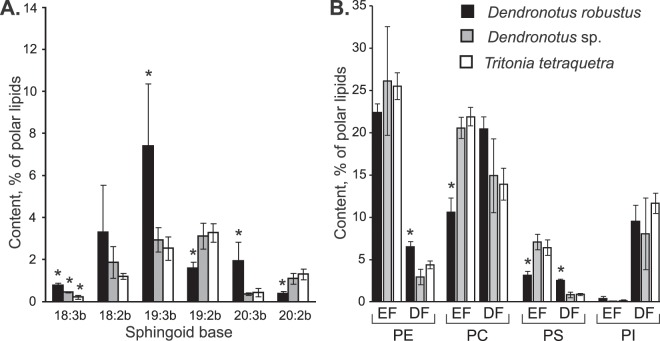
Distribution of ceramide aminoethylphosphonate and structural forms of glycerophospholipids. Comparison of the content of (**A**) ceramide aminoethylphosphonate (CAEP) molecular species (% of polar lipids, mean ± SD) and (**B**) ether (EF) and diacyl forms (DF) of ethanolamine, choline, serine, and inositol glycerophospholipids (PE, PC, PS, and PI, respectively) of three nudibranch molluscs: *Dendronotus robustus* (*n* = 3, black bars), *Dendronotus* sp. (*n* = 3, grey bars), and *Tritonia tetraquetra* (*n* = 7, white bars). Each CAEP molecular species contain *N*-acyl group 16:0 and sphingoid base X:Yb with X carbon atoms and Y double bonds. *Values are significantly different (one-way ANOVA, p < 0.01).

Six CAEP molecular species differed in sphingoid bases, but only palmitic acid (16:0) was detected as *N*-acyl groups of their molecules (Fig. 2, Supplementary Table S4, Supplementary Fig. S5). The set of CAEP molecular species were similar between all the nudibranchs. The considerable amounts of CAEP(19:3b/16:0) in *D. robustus* distinguished it from the other nudibranch species (F_2,16_ = 12.89, p = 0.0017). No significant differences (p > 0.01) in concentration of most CAEP molecular species were observed between *T. tetraquetra* and *Dendronotus* sp. (Supplementary Table S4).

**Figure 3:**
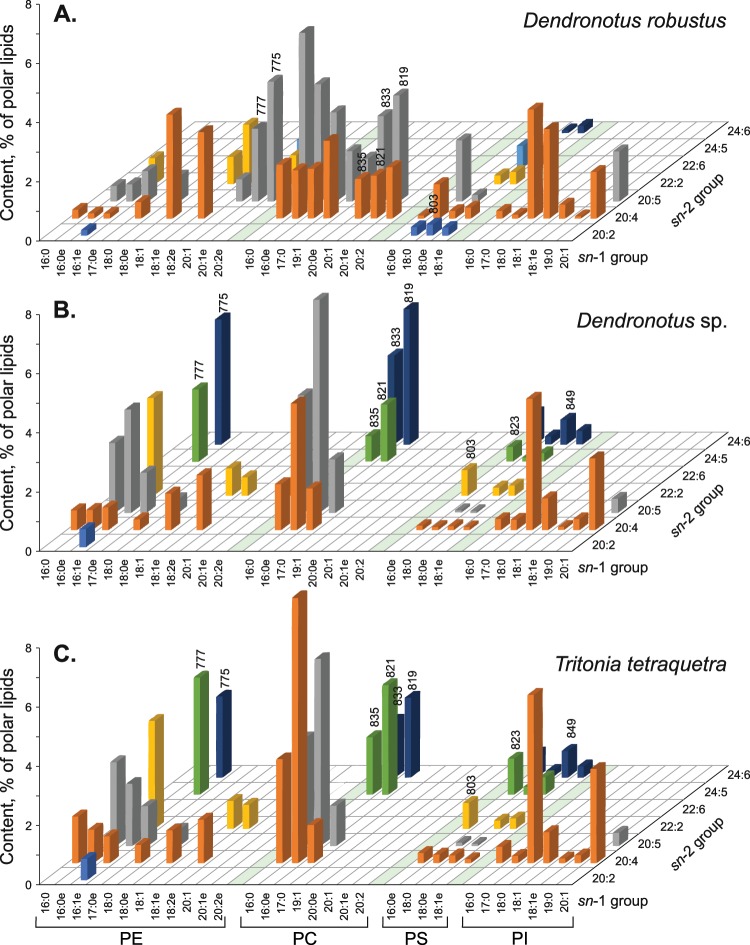
Comparison of polar lipidomes. Average content of major molecular species (% of polar lipids, mean ± SD) of ethanolamine, choline, serine, and inositol glycerophospholipids (PE, PC, PS, and PI, respectively) was compared in three nudibranch molluscs, (**A**) *Dendronotus robustus*, (**B**) *Dendronotus* sp., and (**C**) *Tritonia tetraquetra*. The axis “*sn*-1 group” and axis “*sn*-2 group” indicates alkyl and acyl groups presumably esterified a glycerol backbone of glycerophospholipid molecules in *sn*-1 and *sn*-2 positions, respectively. Alkyl groups (X:Ye) and acyl group (X:Y) contain X carbon atoms and Y double bonds. Bar top numbers indicate molecular weights of major isobaric molecular species.

Both ether (alkylacyl) and diacyl forms were identified in each GPL class (Fig. 3). In all nudibranchs studied, the ether form dominated the PE molecular species, while the diacyl form dominated the PI molecular species. The significantly lower content of the ether form (F_2,16_ = 280.65, p < 0.0001) and the higher content of the diacyl form (F_2,16_ = 7.41, p = 0.01) distinguished *D. robustus* from the other nudibranchs.

**Figure 4:**
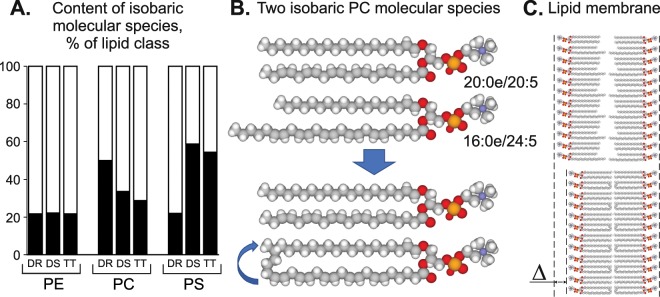
Isobaric molecular species. (**A**) Comparison of the part of isobaric molecular species (% of lipid class, black bars) of ethanolamine glycerophospholipids (PE) with molecular weight 775 and 777, choline glycerophospholipids (PC) with molecular weight 819, 821, 833, and 835, as well as serine glycerophospholipids (PS) with molecular weight 801, 803, 821, 823, and 849, in three nudibranch molluscs, *Dendronotus robustus* (DR), *Dendronotus* sp. (DS), and *Tritonia tetraquetra* (TT). (**B**) Plane scheme of the chemical structures of two PC isobaric molecular species (20:0e/20:5 and 16:0e/24:5; element color: C, gray; H, white, O, red; P, orange; N, blue). (**C**) Illustration of the normalization of lipid membrane thickness, when a shorter alkyl chain may provide a space into lipid bilayer to pack a longer FA chain.

Figure 4 shows a comparison of the profiles of major GPL molecular species between two *Dendronotus* species and *T. tetraquetra*. The percentage of each GPL molecular species in total PL of *T. tetraquetra* were calculated in the present study (Supplementary Table S4) using the molecular species composition of each GPL class and the content of the GPL classes in total PL published earlier^21^. Approximately a half of the polar lipidome of each nudibranch consist of the same GPL molecular species. As can be seen, *T. tetraquetra* is closer to *Dendronotus* sp. than *D. robustus* according to the polar lipidome profile (Fig. 4). The major differences in qualitative composition of GPL molecular species were detected for PE, PC, and PS but not in PI. A considerable amount of the GPL molecular species with TPA acyl groups distinguished *T. tetraquetra* and *Dendronotus* sp. from *D. robustus*. At the same time, *D. robustus* contained a higher relative amount of the GPL molecular species with 22:6n-3 and 20:2NMI than *Dendronotus* sp. and *T. tetraquetra*.

An analysis of PE molecular species showed that *T. tetraquetra* and *Dendronotus* sp. contained 16:1e/24:6 and 16:1e/24:5, which were not found in *D. robustus*. Two other PE molecular species with the same exact molecular weights were detected in *D. robustus*: 20:2e/20:5 instead of 16:1e/24:6 and 20:1e/20:5 instead of 16:1e/24:5 (Fig. 4). These isobaric molecular species had the same element composition, belonged to the same GPL class, had the same form, but differed in the length (and sometimes unsaturation) of alkyl and acyl groups. The isobaric molecular species were also found in PC and PS of the nudibranchs (Fig. 4, Supplementary Table S4). *D. robustus* contained PC(20:1e/20:5), PC(20:0e/20:5), PC(20:1e/20:4), PC(20:1/20:5), PC(20:2/20:4), and PC(20:1/20:4) rather than PC(16:0e/24:6), PC(16:0e/24:5), PC(16:0/24:6), and PC(16:0/24:5) detected in *T. tetraquetra* and *Dendronotus* sp. Three molecular species, (PS(16:1e/22:2), PS(16:0e/22:2), and PS(16:0e/24:5)) were present in *T. tetraquetra* and *Dendronotus* sp. instead of three isobaric molecular species (PS(18:1e/20:2), PS(18:0e/20:2), and PS(18:0e/22:5)) in *D. robustus*. Thus, 20–60% of PE, PC, and PS of the nudibranchs were comprised of the isobaric molecular species (Fig. 4A).

In addition to isobaric molecules, some species-specific lipid compounds were found. Several molecular species of PE (18:0/22:6, 20:1/20:5, 20:2e/22:6), PC (19:1/20:5, 19:2/20:4), and PS (18:0/20:2, 18:1e/22:6) were detected only in *D. robustus* (Fig. 4, Supplementary Table S4). Two diacyl PS molecular species (18:0/24:6 and 18:0/24:5) were observed only in *Dendronotus* sp. and *T. tetraquetra*. These species-specific lipids, which did not have isobaric twins, formed a small part (less than 10%) of each GPL class.

## Discussion

TPA (24:5n-6 and 24:6n-3) are known as chemotaxonomic markers of soft corals and other octocorals^24,25^. According to the concept of FA trophic markers^11^, TPA can be transferred from soft corals to nudibranch molluscs preying on these corals, and, therefore, they may be used to confirm nudibranchs’ trophic relationships. In cold-water benthic ecosystem of the Kuril Islands (the Sea of Okhotsk), the transfer of TPA has been confirmed for the nudibranch mollusc *T. tetraquetra* preying on soft corals^16^. Two cold-water coral species, *Gersemia fruticosa* and *Acanella* sp., were suggested as a major food source of *T. tetraquetra* based on the similarity of the 24:5n-6/24:6n-3 ratio (1.0‒1.4) in *Tritonia* and these corals^16^.

The high level of TPA (24.8%) in total FAs of *Dendronotus* sp. also indicates a significant dietary input of soft corals. *Dendronotus* sp. differed from *T. tetraquetra* by the dominance of 24:6n-3 in TPA (the 24:5n-6/24:6n-3 ratio = 0.16). Hence, another soft coral species is suggested to be a prey of *Dendronotus* sp. Among cold-water soft corals collected in the region of our studies, only species of the family Primnoidae contained trace amounts of 24:5n-6 with a high percentage of 24:6n-3 (9.5‒31.0% of total FAs)^26^. We suppose that the nudibranch *Dendronotus* sp. preys mainly on the Primnoidae soft corals.

In contrast, the low level of TPA (1.4%) in total FAs of *D. robustus* is the evidence of a minor role of soft corals (both aclyonarians and gorgonarians) or other octocorals (for example, sea pens of the Pennatulacea^27^) in the diet of this nudibranch species. Besides octocorals, a number of other benthic invertebrate species, such as bryozoans, hydroids, sponges, amphipods, polychaetas, and scleractinian corals, have also been reported as items of nudibranchs’ diet^2,4,5,28^. Sponges should be removed from the list of possible food of *D. robustus* because no FA markers of this taxon (for example, 26:2 ∆5,9 or 26:3∆5,9,19)^29^ are present in total FA of the species studied. Cold-water scleractinian corals, such as, for example, *Lophelia pertusa*, have not been found in the study region^30^. Campanularid hydroids and oweniid polychaetas were described as food of *D. robustus* from the White Sea and the Barents Sea^3^. Cold-water hydroids, polychaetes, and bryozoans contain relatively low level of 20:4n-6 but considerable amounts of 20:5n-3 and 22:6n-3^26,31,32^, presumably ingested through feeding on microalgae^11^. The nudibranch *D. robustus* showed the proportion of 20:4n-6, 20:5n-3, and 22:6n-3 similar to that in the group of marine invertebrates mentioned above. Hence, some species of this group may be considered as a possible prey of *D. robustus*.

The trophic specialization of several related species inhabiting the same biotope generally reduces the competition between these species and increases the biodiversity and stability of the ecosystem^33^. The method of FA trophic markers confirmed the coexistence of three nudibranch species with different feeding strategies in a cold-water benthic community of the Sea of Okhotsk. Each cold-water nudibranch species consumes specific FAs of prey, which substantially modify the predators’ original FA composition. A similarity of FA profiles of the species within one genus and considerable differences in FA profiles between families have been reported for many marine invertebrates, such as corals and hydrocorals^25,26^. In the present study, the FA composition shows that feeding on octocorals differs *Dendronotus* sp. from *D. robustus* (the family Dendronotidae) but bring *Dendronotus* sp. to the species from another genus and family (*Tritonia*, Tritoniidae). Earlier, large variations of total FA within tropical nudibranchs have been demonstrated^15^. Based on a common analysis of FA composition of total lipids, we may conclude that “nudibranchs are what they eat”. Nudibranchs cannot probably remove or degrade unusual dietary FAs and, thus, have to use these acids in lipid biosynthesis. This makes FAs important markers for identifying nudibranch food sources.

Total FAs are obtained by chemical degradation of total lipids, and, in fact, FAs are parts of original lipid molecules. Total lipids consist of a number of lipid classes, and each lipid class includes a lot of individual lipid molecules, or lipid “molecular species”. The molecular species of one lipid class have the same polar part but different nonpolar alkyl/acyl groups. Lipidome is the entire spectrum of lipids in a biological system^34^, whereas the spectrum of PL molecular species may be defined as polar lipidome. Thus, to estimate actual differences in the structural PL of marine invertebrates, we need to choose the expensive lipidomic analysis rather than the cheap analysis of FAs. For example, the recent studies of kleptoplasty of the sea slug *Elysia viridis*^35^ and polar lipids dynamics during embryonic development of crabs^36^ were based on lipidomic analysis.

In contrast to the sharp differences in the FA profiles, the differences in the TL class composition between the three cold-water nudibranchs were substantially smaller. A comparison of the lipid class profiles confirmed the similarity between *Dendronotus* sp. and *T. tetraquetra*, both preying on octocorals. *D. robustus* can be distinguished from the two other species by the higher proportion of WE, which are a long-term energy reserve and vary depending on species and environmental factors^11,37^. The cold-water species contained a moderate part (6–18% of TL) of storage lipids (WE and TG), but the content of the storage lipids in TL of the earlier described tropical species^15^ was even lower. Structural PL dominated TL of both the cold-water species studied and the tropical species^15^. The PL class distribution was found to be quite similar within the cold-water species. The same can be observed within tropical species^15^. Hence, the profile of PL classes seems to be specific for each geographic region. Despite the variety of food sources, nudibranchs maintain a relatively constant PL class composition most suitable for their habitat. A stabile composition of structural PL classes is required for normal biomembrane functioning^38–40^.

In contrast to mammalian lipids, which mainly contain diacyl GPL^40^, lipids of molluscs are characterised by a large part of ether GPL (plasmalogen and alkylacyl GPL)^41^. Synthesis of both diacyl and ether GPL begins in peroxisomes, where fatty acyl-CoA is utilized by two different enzymes, forming acyl and alkyl dihydroxyacetone phosphates (acyl- and alkyl-DHAP)^40^. Pathways from acyl-DHAP to diacyl GPL and from alkyl-DHAP to ether GPL are completed in endoplasmic reticulum^42^. A preferential formation of acyl-DHAP may explain the higher level of diacyl GPL and the lower level of ether GPL, which distinguished *D. robustus* from *Dendronotus* sp. and *T. tetraquetra*. This effect refers to PE, PC, and PS but not to PI. Similarly to bivalves and gastropods^41^, nudibranch molluscs contain PI in the diacyl form with trace amounts of ether forms. As a lipidomic analysis shows, neither dietary FAs (24:5n-6, 24:6n-3, and 22:6n-3) nor FA markers of molluscs (20:2NMI and 22:2NMI) are used by the cold-water nudibranchs for synthesis of PI. Hence, PI, as well as CAEP, may be considered as a conservative part of the nudibranch polar lipidome, which is not influenced by dietary lipids. Functions of PI in molluscs are still unclear. In mammal tissues, PI is the source of arachidonic acid required for biosynthesis of eicosanoids and the source of diacylglycerols that act as signalling molecules; PI and polyphosphoinositides have crucial significance in interfacial binding of proteins^42^. Possibly, some of these functions require maintaining a relatively constant set of PI molecular species in the cold-water nudibranchs.

A comparison of *D. robustus* with *Dendronotus* sp. and *T. tetraquetra* preying on octocorals showed that variations in the dietary FA composition lead to a diversity of the molecular species of PE, PC, and PS in the nudibranchs. A lipidomic analysis of these GPL classes detected an intriguing feature of the qualitative composition of their molecular species. The nudibranchs with different food sources had a different set of the isobaric molecular species, which belonged to the same GPL class, had the same ether form, but differed in the length of alkyl and acyl groups. Such isobaric molecular species contained alkyl/acyl groups C_20_/C_20_ and C_18_/C_22_, when C_24_ TPA were absent from dietary lipids, or contained C_16_/C_24_ groups, when dietary C_24_ TPA were presented (Fig. 4B). Moreover, *D. robustus* contained PS(18:1e/20:2) and PS(18:0e/20:2), while other nudibranchs contained isobaric PS(16:1e/22:2) and PS(16:0e/22:2).

Hence, the cold-water nudibranchs are capable of reducing the length of alkyl group of PE, PC, and PS molecular species in response to the increase in the length of acyl group. The length of alkyl/acyl group of PL molecules strongly influences the thickness of lipid bilayer, which is critical for proper functioning of membranes. A shorter alkyl chain seems to provide vacant space to pack a longer FA chain into lipid bilayer (Fig. 4B,C). This phenomenon is suggested as a possible molecular mechanism of adaptation of nudibranch polar lipidomes to the dietary lipid input.

In the cold-water nudibranchs studied, the polar lipidome is order-specific and much less dependent on the dietary lipid input than total FAs. Despite amounts of the storage lipids vary, the composition of the structural lipids remains relatively stable. The nudibranchs do not reject or decompose dietary very-long-chain FAs. In response to variation in dietary FA length, the polar lipidome and membrane structure is adjusted by the synthesis of isobaric PE, PC, and PS molecular species. We suppose that this feature may explain the amazing ability of nudibranch molluscs consume a wide variety of food sources^2,3,5^, including sponges with unusual C_24-30_ FAs^28,43^.

## Methods

### Chemicals

The using of chemicals was described previously^21^. All solvents were of HPLC or LCMS grade. Polar lipid standards (16:0-20:4 PC, 16:0-20:4 PE, 16:0-20:4 PS, 18:0-20:4 PI, C18(Plasm)-20:4 PC, C18(Plasm)-20:4 PE, and C16-18:1 PC) were from Avanti Polar Lipid Co. (Alabaster, USA). Neutral lipid standards (stearyl oleate, cholesterol oleate, 1-*O*-hexadecyl-2,3-dihexadecanoyl-*rac*-glycerol, glycerol trioleate, oleic acid, and cholesterol), a mixture of PUFA methyl esters No. 3 from menhaden oil, and column silica gel (high-purity grade, 70-230 mesh) were purchased from Sigma-Aldrich Co. (St. Louis, USA). The silica gel TLC plates (PTLC-AF-V) with a silica sol binder on aluminium foil were provided by Sorbfil (Krasnodar, Russian Federation).

### Collection of specimens

The nudibranch molluscs of the genus *Dendronotus* Alder & Hancock, 1845 (*Dendronotus robustus* A. E. Verrill, 1870 and *Dendronotus* sp.) were collected with a dredge off the islands Iturup and Urup (Kuril Islands, 45°15′−46°16′ N, 147°24′−150°15′ E) at a depth of 120–200 m in July 2011. Six specimens of each nudibranch species were used for a TL, PL, and FA analysis, three specimens were used for PL molecular species analysis.

### Lipid extraction

Total lipids were extracted from fresh animal samples immediately aboard the vessel. The extraction technique of Folch *et al*.^44^ was modified according to Imbs *et al*.^21^. The samples were homogenized in a chloroform:methanol (1:2, *by vol*.) mixture (30 mL per 10 g wet weight). The obtained homogenate was filtered, and the residue was repeatedly extracted (6 h, 4 °C) in a chloroform:methanol (2:1, *by vol*.) mixture (2 × 30 mL). The extracts were then mixed and separated into layers by adding 35 mL of water and 30 mL of chloroform. The lower layer was separated and evaporated. The total lipids were dissolved in chloroform and stored at −80 °C.

### Lipid class analysis

Lipid classes were separated by TLC according to Imbs and Chernyshev^21^. Each sample was placed on two TLC plates (10 × 10 cm). For total lipid analysis, one plate was first developed to its full length with hexane:diethyl ether:acetic acid (70:30:1, *by vol*.) and finally to 25% length with chloroform: MeOH:28% NH_4_OH (65:35:5, *by vol*.) (Supplement Figure S1A). For polar lipid analysis, the second plate was developed with the last solvent system (Supplement Fig. S1B). After drying in an air stream, the plates were sprayed with 10% H_2_SO_4_/MeOH and heated at 240 °C for 10 min. The chromatograms were scanned with an Epson Perfection 2400 PHOTO image scanner (Nagano, Japan) in a grayscale mode. Percentages of lipid contents were calculated based on band intensity values using Sorbfil TLC Videodensitometer image analysis program (Krasnodar, Russia). Units were calibrated with the use of standards for each lipid class as described previously^45^.

### Fatty acid analysis

Fatty acid methyl esters (FAME) were obtained by a treatment of the total lipids with 2% H_2_SO_4_/MeOH at 80 °C for 2 h in a screw-caped vial under argon, extracted with hexane and purified by preparative TLC developed in benzene. 4,4-Dimethyloxazoline (DMOX) derivatives of FA were prepared according to Svetashev^46^. A gas chromatography analysis of FAME was conducted with a GC-2010 chromatograph (Shimadzu, Kyoto, Japan) with a flame ionization detector. A Equity-5 (Supelco, Bellefonte, USA) capillary column (30 m × 0.25 mm i.d., film thickness 25 µm) was held for 2 min at 170 °C, then heated with a 2 °C min^−1^ ramp to 240 °C that was held for 5 min. The injector (250 °C) and detector (260 °C) temperatures were constant. Helium was used as the carrier gas at a linear velocity of 30 cm s^−1^. Identification of FAs was confirmed by GC−MS of their methyl esters and DMOX derivatives on a GCMS-2010 Ultra instrument (Shimadzu, Kyoto, Japan) (electron impact at 70 eV) and a MDN-5s (Supelco, Bellefonte, USA) capillary column (30 m × 0.25 mm ID). Carrier gas was He at 30 cm s^−1^. The GC−MS analysis of FAME was performed at 160 °C with a 2 °C min^−1^ ramp to 240 °C that was held for 20 min. The injector and detector temperatures were 250 °C. GC−MS of DMOX derivatives was performed at 210 °C with a 3 °C min^−1^ ramp to 270 °C that was held for 40 min. The injector and detector temperatures were 270 °C. Spectra were compared with the NIST library and FA mass spectra archive^47,48^.

### Analysis of lipid molecular species

Analysis was performed according to Imbs and Chernyshev^21^. Separation of lipids was performed on a Shimadzu Prominence liquid chromatograph equipped with two LC-20AD pump units, a high-pressure gradient forming module, a CTO-20A column oven, a SIL-20A auto sampler, a CBM-20A communications bus module, a DGU-20A 3 degasser, and a Shim-Pack diol column (50 mm × 4.6 mm ID, 5 μm particle size) (Shimadzu, Kyoto, Japan). Lipid samples and authentic standards were eluted with a binary gradient of (A) hexane:2-propanol:AcOH:Et_3_N (82:17:1:0.08, *by vol*.) and (B) 2-propanol:H_2_O:AcOH:Et_3_N (85:14:1:0.08, *by vol*.). The gradient started at 5% of mixture B and its percentage increased to 80% over 25 min. This composition was maintained for 1 min, returned to 5% of mixture B over 10 min, and maintained at 5% for 4 min (the total run time was 40 min). The flow rate was 0.2 mL min^−1^. The column was held at 40 °C. The injection volume was 10 µL. The eluent outlet was connected to a MS analyser. Lipids were detected by a high resolution tandem ion trap–time of flight mass spectrometry with a Shimadzu LCMS-IT-TOF instrument (Kyoto, Japan) operating both at positive and negative ion mode during each analysis at electrospray ionization (ESI) conditions. The ion source temperature was 200 °C, the range of detection was 100–1200 *m/z*, and potential in the ion source was −3.5 for negative mode and 4.5 kV for positive mode. The drying gas (N_2_) pressure was 200 kPa. The nebulizer gas (N_2_) flow was 1.5 L min^−1^. The acquisition of MS data was processed using the mass spectrometer instrument software (Shimadzu LCMSSolution (LCMS-IT-TOF) v. 3.50). The external phospholipid standards were used to determine a retention time of each GPL class and to check fragmentation pathways of the GPL classes during tandem mass spectrometry experiments. The peaks of ions [M−H]^−^ were used for detection and quantification of molecular species of CAEP, PE, PS, and PI, but more intensive peaks of ions [M + CH_3_COO]^−^ were selected for PC molecular species. The element composition and number of double bonds of each ion peak within the range of each PL class was manually calculated using the mass spectrometer instrument software. For the ions with the element composition corresponding to certain PL classes, tandem mass spectrometry experiments were subsequently conducted in the positive and negative modes to acquire the product ion spectrum of each PL molecular species. The chemical structure of the PL molecular species was identified as described earlier (Supplementary Figs. S5–S9)^21,22,49^. Quantification of individual molecular species within each PL class was carried out by calculating the peak areas for the individual extracted ion chromatograms. The ion chromatogram of each even nominal *m/z* was manually extracted from the total ion chromatogram of each sample. After the latent isotope peaks were removed, the list of the peak areas of each PL class was exported as a Microsoft Excel file for further analysis. The percentages of the individual molecular species in total PL were calculated using the molecular species composition of each PL class and the content of each PL class in total PL previously quantified by TLC (Supplementary Table S2).

### Statistical analysis

The analysis approach was recently described^22^. Briefly, significance of differences in mean contents of FAs, lipid classes, and lipid molecular species between nudibranch species was tested by one-way analysis of variance (ANOVA). Raw data were used following evaluation of the homogeneity of variances (Levene’s test) and the normality of data distribution (Shapiro–Wilk test). To represent differences between nudibranch species, the variables (square roots of TL class, or PL class, or FA contents) were included in principal components analyses (PCA). All statistical analyses were performed using STATISTICA 5.1 (StatSoft, Inc., USA). A statistical probability of p < 0.01 was considered significant. Values are represented as mean ± standard deviation.

## Supplementary information

Supplementary Information.
