# Supplementary material for: Lipidomic study of the influence of dietary fatty acids on structural lipids of cold-water nudibranch molluscs
Source: Sci Rep. 2019 Dec 27;9:20013. doi: 10.1038/s41598-019-56746-8 (PMC6934475; doi:10.1038/s41598-019-56746-8)
Supplement: Supplementary file 1 — Supplementary Information. [file 41598_2019_56746_MOESM1_ESM.pdf]

Supplementary information

Lipidomic study of the influence of dietary fatty acids on structural lipids of cold-water nudibranch molluscs

Andrey B. Imbs & Valeria P. Grigorchuk

Supplementary Table S1. Total lipid composition (% of total lipids, mean ± SD) of three nudibranch molluscs from the Sea of Okhotsk.

Lipid class	*Dendronotus robustus* *n* = 6	*Dendronotus* sp. *n* = 6	*Tritonia tetraquetra** *n* = 7	$F_{2,16}$	p**
Polar lipids (PL)	56.2 ± 3.1[a,b]***	64.0 ± 4.7[a]	62.5 ± 2.8[b]	8.10	0.0037
Sterols (ST)	20.4 ± 3.4	22.9 ± 3.6	17.3 ± 2.5	5.13	0.0190
Free fatty acids (FFA)	1.0 ± 0.3	1.7 ± 0.4	2.0 ± 1.7	1.37	0.2813
Triacylglycerols (TG)	0.7 ± 0.2[a,b]	2.2 ± 0.4[a]	3.0 ± 1.8[b]	6.40	0.0091
Sterol esters (SE)	1.6 ± 0.7	1.4 ± 1.2	2.8 ± 0.9	3.98	0.0394
Wax esters (WE)	17.9 ± 4.9[a,b]	3.0 ± 1.5[a,c]	8.8 ± 1.4[b,c]	37.47	>0.0001

* Imbs, A. B. High level of tetracosapolyenoic fatty acids in the cold-water mollusk *Tochuina tetraquetra* is a result of the nudibranch feeding on soft corals. Polar Biology 39, 1511-1514 (2016), doi:10.1007/s00300-015-1865-y.

** A statistical probability of $p < 0.01$ was considered significant.

*** Mean values marked by the same letters differ significantly.

Supplementary Table S2. Polar lipid composition (% of polar lipids, mean ± SD) of three nudibranch molluscs from the Sea of Okhotsk.

Polar lipid class	*Dendronotus robustus* *n* = 6	*Dendronotus* sp. *n* = 6	*Tritonia tetraquetra** *n* = 7	$F_{2,16}$	p**
Ethanolamine glycerophospholipids (PE)	33.5 ± 2.0	34.8 ± 3.6	31.1 ± 7.1	1.05	0.3724
Choline glycerophospholipids (PC)	31.6 ± 2.5	34.3 ± 2.4	35.8 ± 3.7	3.64	0.0498
Serine glycerophospholipids (PS)	5.7 ± 0.9[a,b***]	7.4 ± 0.3[a]	7.2 ± 1.3[b]	7.08	0.0063
Inositol glycerophospholipids (PI)	10.1 ± 1.3	11.5 ± 1.2	11.8 ± 1.5	3.15	0.0704
Ceramide aminoethylphosphonate (CAEP)	15.3 ± 1.0[a,b]	9.6 ± 1.3[a]	8.9 ± 1.0[b]	61.30	>0.0001

*, **, *** Ref. Supplementary Table S1.

Supplementary Table S3. Fatty acid composition (% of total fatty acids, mean ± SD) of total lipids of three nudibranch molluscs from the Sea of Okhotsk.

Fatty acid (FA)	*Dendronotus robustus* *n* = 6	*Dendronotus* sp. *n* = 6	*Tritonia tetraquetra** *n* = 7	$F_{2,16}$	p**
14:0	0.6 ± 0.2	0.6 ± 0.2	0.5 ± 0.1	0.67	0.5241
15:0	0.4 ± 0.1[a***]	0.6 ± 0.1[a,b]	0.3 ± 0.1[b]	12.12	0.0006
i-16:0	0.2 ± 0.1[a]	0.4 ± 0.1[a,b]	0.1 ± 0.0[b]	17.45	0.0001
16:1n-10	0.4 ± 0.1	0.3 ± 0.2	0.3 ± 0.1	1.08	0.3623
16:1n-7	0.4 ± 0.2[a,b]	1.2 ± 0.2[a]	1.3 ± 0.3[b]	18.75	0.0001
16:1	0.3 ± 0.1[a,b]	0.2 ± 0.1[a]	0.1 ± 0.0[b]	8.67	0.0028
16:0	10.4 ± 1.5	10.6 ± 0.7	9.9 ± 1.2	0.54	0.5939
br-17:1	0.0 ± 0.1[a,b]	0.2 ± 0.1[a]	0.2 ± 0.0[b]	12.94	0.0005
7-Me-16:1n-10	2.7 ± 0.5[a,b]	0.9 ± 0.2[a]	0.8 ± 0.2[b]	65.16	>0.0001
i-17:0	0.9 ± 0.2[a]	0.9 ± 0.1[b]	0.5 ± 0.1[a,b]	24.78	>0.0001
ai-17:0	0.7 ± 0.1[a,b]	0.4 ± 0.1[a,c]	0.2 ± 0.1[b,c]	25.16	>0.0001
17:0	0.3 ± 0.1[a,b]	0.6 ± 0.1[a,c]	0.5 ± 0.1[b,c]	16.14	0.0001
br-18:1	0.0 ± 0.1[a,b]	0.3 ± 0.1[a]	0.2 ± 0.1[b]	17.97	>0.0001
i-18:0	2.1 ± 0.5[a,b]	0.3 ± 0.1[a]	0.2 ± 0.1[b]	79.90	>0.0001
18:2n-6	0.9 ± 0.3	0.8 ± 0.4	0.6 ± 0.3	1.21	0.3245
18:1n-9	3.1 ± 1.8	3.0 ± 0.7	3.4 ± 0.8	0.18	0.8404
18:1n-7	1.0 ± 0.2	0.7 ± 0.3	1.1 ± 0.2	4.97	0.0209
18:1	0.5 ± 0.2	0.4 ± 0.1	0.4 ± 0.0	0.56	0.5804
18:0	12.0 ± 3.8[a,b]	4.6 ± 0.2[a]	5.0 ± 0.3[b]	23.18	>0.0001
19:1	0.2 ± 0.0[a,b]	0.1 ± 0.1[a]	0.1 ± 0.0[b]	8.08	0.0038
19:0	0.1 ± 0.0	0.1 ± 0.0	0.1 ± 0.0	0.84	0.4490
20:4n-6	7.4 ± 0.9[a,b]	13.8 ± 2.8[a,c]	22.7 ± 1.8[b,c]	99.58	>0.0001
20:5n-3	21.2 ± 2.6[a,b]	17.6 ± 3.3[a,c]	13.5 ± 1.7[b,c]	14.79	0.0002
20:2NMI	2.8 ± 1.1[a,b]	1.7 ± 1.0[a]	1.0 ± 0.4[b]	7.43	0.0052
20:2NMI	0.1 ± 0.1[a]	0.2 ± 0.1[b]	0.4 ± 0.1a,b	10.09	0.0015
20:2n-6	0.1 ± 0.1[a]	0.1 ± 0.1[b]	0.1 ± 0.1a,b	0.71	0.5072
20:1n-11	0.0 ± 0.0	0.1 ± 0.1	0.3 ± 0.1	22.38	>0.0001
20:1n-9	7.2 ± 2.0[a,b]	3.5 ± 1.4[a]	2.2 ± 0.8[b]	20.26	>0.0001
20:1n-7	4.1 ± 1.1[a,b]	1.2 ± 0.4[a,c]	2.2 ± 0.6[b,c]	24.16	>0.0001
22:6n-3	12.1 ± 2.4[a,b]	0.7 ± 0.2[a]	0.5 ± 0.2[b]	150.01	>0.0001

22:4n-6	0.5 ± 0.1^{a}	0.4 ± 0.1^{b}	0.9 ± 0.3^{a,b}	12.34	0.0006
22:5n-3	1.8 ± 0.3^{a,b}	0.6 ± 0.2^{a}	0.7 ± 0.1^{b}	64.62	>0.0001
7,13-22:2	2.2 ± 0.1^{a,b}	6.1 ± 1.8^{a}	5.0 ± 1.1^{b}	17.95	0.0001
7,15-22:2	0.2 ± 0.1^{a,b}	0.9 ± 0.3^{a,c}	1.6 ± 0.5^{b,c}	26.63	>0.0001
24:5n-6	0.3 ± 0.1^{a,b}	3.4 ± 0.6^{a,c}	10.5 ± 2.4^{b,c}	80.44	>0.0001
24:6n-3	1.1 ± 0.4^{a,b}	21.4 ± 1.7^{a,c}	10.7 ± 1.5^{b,c}	333.71	>0.0001
Other FA	1.9 ± 0.6	1.4 ± 0.3	2.0 ± 0.2	-	-
SFAs***	27.6 ± 4.9^{a,b}	19.1 ± 0.8^{a}	17.3 ± 1.5^{b}	22.11	>0.0001
MUFAs	17.2 ± 4.4^{a,b}	11.0 ± 1.5^{a}	11.7 ± 1.6^{b}	8.84	0.0026
PUFAs	55.3 ± 4.8^{a,b}	69.9 ± 2.0^{a}	71.0 ± 2.9^{b}	40.49	>0.0001
n-6 PUFAs	9.1 ± 0.8^{a,b}	18.4 ± 3.0^{a,c}	34.6 ± 3.5^{b,c}	146.08	>0.0001
n-3 PUFAs	36.3 ± 3.8^{a}	40.3 ± 3.7^{b}	25.5 ± 2.2^{a,b}	36.39	>0.0001
n-6/n-3 PUFA	0.3 ± 0.0^{a}	0.5 ± 0.1^{b}	1.4 ± 0.2^{a,b}	108.31	>0.0001
i+ai+br	6.6 ± 0.5^{a,b}	3.3 ± 0.3^{a,c}	2.2 ± 0.5^{b,c}	166.59	>0.0001
NMI FAs	5.2 ± 1.0^{a,b}	8.8 ± 2.3^{a}	8.0 ± 1.2^{b}	8.44	0.0031
C_{24} PUFAs	1.4 ± 0.4^{a,b}	24.8 ± 2.0^{a}	21.2 ± 3.8^{b}	147.33	>0.0001

*, **, *** Ref. Supplementary Table S1.

**** SFAs, saturated FAs; MUFAs, monounsaturated FAs; PUFAs, polyunsaturated FAs; n-6 PUFA, the sum of n-6 series PUFAs; n-3 PUFA, the sum of n-3 series PUFAs; n-6/n-3 PUFA, n-6/n-3 PUFA ratio; i+ai+br, the sum of iso-, antiso-, and other branched FAs; NMI FAs, non-methylene-interrupted FAs; C_{24} PUFAs, the sum of tetracosapolyenoic FAs.

Supplementary Table S4. Molecular species composition of ceramide aminoethylphosphonate (CAEP), ethanolamine glycerophospholipids (PE), choline glycerophospholipids (PC), serine glycerophospholipids (PS), and inositol glycerophospholipids (PI) (% of polar lipids, mean ± SD) of three nudibranch molluscs from the Sea of Okhotsk.

Lipid molecular species	Measured value (*m/z*)	Predicted value (*m/z*)	Mass error (ppm)	*Dendronotus robustus* *n* = 3	*Dendronotus* sp. *n* = 3	*Tritonia tetraquetra** *n* = 7	$F_{2,16}$	p**
**CAEP**	$[M-H]^-$	$[M-H]^-$						
18:3b/16:0	639.4833	639.4871	5.98	0.77 ± 0.11[a,b***]	0.45 ± 0.02[a,c]	0.21 ± 0.09[b,c]	47.80	>0.0001
18:2b/16:0	641.4998	641.5028	4.64	3.30 ± 2.25	1.86 ± 0.76	1.20 ± 0.13	4.04	0.0517
br-19:3b/16:0	653.4979	653.5028	7.46	7.40 ± 2.98[a,b]	2.94 ± 0.58[a]	2.53 ± 0.56[b]	12.89	0.0017
br-19:2b/16:0	655.5149	655.5184	5.38	1.59 ± 0.29[a,b]	3.12 ± 0.61[a]	3.27 ± 0.44[b]	15.05	0.0010
20:3b/16:0	667.5137	667.5184	7.08	1.93 ± 0.89[a,b]	0.37 ± 0.05[a]	0.43 ± 0.20[b]	14.56	0.0011
20:2b/16:0	669.5282	669.5341	8.77	0.38 ± 0.11[a,b]	1.09 ± 0.25[a]	1.30 ± 0.26[b]	16.47	0.0006
**PE**	$[M-H]^-$	$[M-H]^-$						
16:1e/20:5****	720.4942	720.4974	4.39	0.58 ± 0.11[a,b]	2.03 ± 0.15[a,c]	2.10 ± 0.47[b,c]	48.60	>0.0001
16:1e/20:4	722.5107	722.5130	3.20	0.17 ± 0.07[a,b]	0.76 ± 0.05[a]	0.91 ± 0.10[b]	77.81	>0.0001
16:0e/20:5	722.5107	722.5130	3.20	0.53 ± 0.21[a,b]	2.37 ± 0.15[a]	2.83 ± 0.31[b]	78.68	>0.0001
16:0e/20:4	724.5242	724.5287	6.15	0.17 ± 0.08[a]	0.68 ± 0.05	1.13 ± 0.31[a]	16.61	0.0006
16:1e/20:2	726.5419	726.5443	3.32	0.19 ± 0.06	0.61 ± 0.15	0.72 ± 0.29	5.45	0.0250
17:0e/20:5	736.5295	736.5287	1.13	1.03 ± 0.61	1.36 ± 0.16	1.35 ± 0.57	0.44	0.6581
16:0/20:4	738.5053	738.5079	3.55	0.30 ± 0.15[a]	0.67 ± 0.17[b]	1.58 ± 0.21[a,b]	52.17	>0.0001
18:2e/20:4	748.5242	748.5287	5.96	2.91 ± 0.28[a,b]	1.85 ± 0.34[a]	1.47 ± 0.31[b]	25.64	0.0001
18:1e/20:4	750.5395	750.5443	6.41	0.65 ± 0.15	0.36 ± 0.07	0.43 ± 0.14	4.66	0.0371

18:0e/20:5	750.5355	750.5443	6.41	0.85 ± 0.20	0.46 ± 0.09	0.56 ± 0.19	4.68	0.0367
16:0e/22:5	750.5355	750.5443	6.41	0.18 ± 0.04	0.10 ± 0.02	0.12 ± 0.04	4.22	0.0468
16:1e/22:2	754.5724	754.5756	4.25	0.88 ± 0.14^{a,b}	3.30 ± 0.83^{a}	3.65 ± 0.79^{b}	15.72	0.0008
18:1/20:4	764.5201	764.5236	4.54	3.51 ± 0.48^{a,b}	1.23 ± 0.04^{a}	1.11 ± 0.16^{b}	104.12	>0.0001
18:0/20:4	766.5354	766.5392	4.99	0.56 ± 0.15	0.37 ± 0.07	0.62 ± 0.09	5.77	0.0216
18:2/20:2	766.5354	766.5392	4.99	0.49 ± 0.13	0.33 ± 0.06	0.54 ± 0.08	4.76	0.0352
16:2/22:2	766.5354	766.5392	4.99	0.42 ± 0.11	0.27 ± 0.06	0.47 ± 0.07	6.87	0.0133
17:1e/22:2	768.5870	768.5913	5.54	0.07 ± 0.03^{a,b}	0.89 ± 0.16^{a,c}	0.36 ± 0.16^{b,c}	26.95	>0.0001
16:1e/24:6	774.5411	774.5443	4.14	-	4.22 ± 0.81^{a}	2.74 ± 0.59^{a}	12.44*****	0.0078
16:1e/24:5	776.5571	776.5600	3.68	-	2.44 ± 0.72	3.96 ± 1.14	3.89*****	0.0840
20:2e/20:5	774.5411	774.5443	4.14	4.05 ± 0.62	-	-	-	-
20:1e/20:5	776.5571	776.5600	3.68	2.45 ± 0.44	-	-	-	-
18:2e/22:2	780.5899	780.5913	1.75	2.02 ± 0.48^{a,b}	0.63 ± 0.17^{a}	0.82 ± 0.39^{b}	13.75	0.0014
18:1e/22:2	782.6036	782.6069	4.23	0.93 ± 0.11	0.93 ± 0.16	0.95 ± 0.61	0.00	0.9996
18:0/22:6	790.5405	790.5392	1.61	0.51 ± 0.05	-	-	-	-
20:1/20:5	790.5405	790.5392	1.61	0.75 ± 0.05	-	-	-	-
20:2e/22:6	800.5577	800.5600	2.82	2.20 ± 0.19	-	-	-	-
20:1e/22:6	802.5725	802.5756	3.88	0.93 ± 0.16^{a,b}	0.62 ± 0.16^{a}	0.44 ± 0.09^{b}	16.25	0.0007
20:2e/22:5	802.5725	802.5756	3.88	0.51 ± 0.09^{a,b}	0.35 ± 0.09^{a}	0.24 ± 0.05^{b}	16.24	0.0007
20:2e/22:2	808.6184	808.6226	5.14	0.98 ± 0.35	0.59 ± 0.25	0.68 ± 0.49	0.85	0.4577
**PC**	$[M+CH_3COO]^-$	$[M+CH_3COO]^-$						
16:0e/20:5	824.5788	824.5811	2.78	3.96 ± 0.45^{a,b}	7.18 ± 0.52^{a,c}	6.31 ± 0.73^{b,c}	33.07	>0.0001
16:0e/20:4	826.5951	826.5967	1.99	1.64 ± 0.32^{a,b}	4.26 ± 0.90^{a,c}	9.14 ± 0.83^{b,c}	97.26	>0.0001

16:0/20:5	838.5576	838.5604	3.29	5.69 ± 1.90	3.96 ± 1.28	3.64 ± 0.84	2.92	0.1005
16:0/20:4	840.5769	840.5760	1.06	1.82 ± 0.26[a]	1.55 ± 0.41[b]	3.50 ± 0.53[a,b]	20.61	0.0003
17:0/20:5	852.5779	852.5760	2.22	3.02 ± 0.59[a,b]	1.81 ± 0.15[a,c]	1.36 ± 0.29[b,c]	24.02	0.0002
17:0/20:4	854.5879	854.5917	4.39	1.69 ± 0.53	1.41 ± 0.22	1.29 ± 0.59	0.81	0.4730
16:0e/24:6	878.6224	878.6280	6.42	-	4.56 ± 0.50[a]	2.69 ± 0.23[a]	113.36	>0.0001
20:1e/20:5	878.6255	878.6280	2.89	3.58 ± 0.70	-	-	-	-
19:1/20:5	878.5949	878.5917	3.69	1.70 ± 0.33	-	-	-	-
19:2/20:4	878.5949	878.5917	3.69	1.06 ± 0.21	-	-	-	-
16:0e/24:5	880.6394	880.6437	4.87	-	1.92 ± 0.26[a]	3.70 ± 0.39[a]	32.83*****	0.0004
20:0e/20:5	880.6398	880.6437	4.42	1.45 ± 0.43	-	-	-	-
20:1e/20:4	880.6398	880.6437	4.42	1.45 ± 0.43	-	-	-	-
19:1/20:4	880.6043	880.6073	3.41	2.63 ± 0.78	-	-	-	-
16:0/24:6	892.6046	892.6073	3.03	-	3.00 ± 0.65[a]	1.92 ± 0.33[a]	22.23*****	0.0015
20:1/20:5	892.6064	892.6073	1.02	2.89 ± 0.29	-	-	-	-
20:2/20:4	892.6064	892.6073	1.02	1.74 ± 0.18	-	-	-	-
16:0/24:5	894.6176	894.6230	5.98	-	0.86 ± 0.14[a]	1.94 ± 0.19[a]	55.91*****	>0.0001
20:1/20:4	894.6220	894.6230	1.07	1.34 ± 0.46	-	-	-	-
**PS**	$[M-H]^-$	$[M-H]^-$						
16:0e/20:4	768.5212	768.5185	3.52	0.10 ± 0.04[a]	0.14 ± 0.05[b]	0.34 ± 0.05[a,b]	31.66	>0.0001
18:0e/20:5	794.5330	794.5341	1.44	0.21 ± 0.03[a,b]	0.05 ± 0.01[a]	0.07 ± 0.02[b]	42.66	>0.0001
18:1e/20:4	794.5330	794.5341	1.44	0.37 ± 0.05[a,b]	0.09 ± 0.02[a]	0.13 ± 0.03[b]	43.23	>0.0001
18:0e/20:4	796.5479	796.5498	2.37	0.26 ± 0.08	0.13 ± 0.01	0.26 ± 0.05	4.49	0.0407
16:1e/22:2	798.5622	798.5654	4.06	-	0.09 ± 0.05	0.12 ± 0.03	0.01*****	0.9146

16:0e/22:2	800.5781	800.5811	3.73	-	0.86 ± 0.28	0.88 ± 0.17	1.97*****	0.1986
18:1e/20:2	798.5677	798.5654	2.82	0.27 ± 0.09	-	-	-	-
18:0e/20:2	800.5837	800.5811	3.25	0.37 ± 0.08	-	-	-	-
18:0/20:5	808.5112	808.5134	2.73	2.06 ± 0.40^{a,b}	0.08 ± 0.02^{a}	0.11 ± 0.02^{b}	134.06	>0.0001
18:0/20:4	810.5266	810.5291	3.03	1.16 ± 0.14^{a,b}	0.11 ± 0.03^{a}	0.29 ± 0.10^{b}	89.48	>0.0001
18:0/20:2	814.5590	814.5604	1.67	0.29 ± 0.04	-	-	-	-
18:1e/22:6	818.5325	818.5341	2.00	0.47 ± 0.13	-	-	-	-
16:0e/24:6	820.5460	820.5498	4.62	-	1.05 ± 0.32	0.81 ± 0.23	7.03*****	0.0292
18:1e/22:5	820.5476	820.5498	2.67	0.07 ± 0.02	-	-	-	-
18:0e/22:6	820.5476	820.5498	2.67	0.72 ± 0.08	-	-	-	-
16:0e/24:5	822.5605	822.5654	6.00	-	0.49 ± 0.03	1.22 ± 0.30	11.00*****	0.0106
18:0e/22:5	822.5646	822.5654	1.02	0.22 ± 0.09	-	-	-	-
18:1e/22:2	826.5921	826.5967	5.61	0.42 ± 0.25	0.34 ± 0.18	0.36 ± 0.10	0.25	0.7839
18:0e/22:2	828.6096	828.6124	3.37	0.30 ± 0.05	0.27 ± 0.08	0.28 ± 0.11	0.59	0.5724
18:1e/24:6	846.5636	846.5654	2.17	0.25 ± 0.08^{a,b}	0.45 ± 0.04^{a,c}	0.38 ± 0.05^{b,c}	29.17	>0.0001
18:1e/24:5	848.5775	848.5811	4.23	0.06 ± 0.03	-	-	-	-
18:0e/24:6	848.5775	848.5811	4.23	0.09 ± 0.04^{a,b}	0.83 ± 0.13^{a}	0.91 ± 0.09^{b}	89.44	>0.0001
20:1e/22:5	848.5775	848.5811	4.23	0.06 ± 0.03	-	-	-	-
18:0e/24:5	850.5949	850.5967	2.16	0.08 ± 0.02^{a,b}	0.28 ± 0.03^{a,c}	0.59 ± 0.06^{b,c}	125.31	>0.0001
18:0/24:6	862.5558	862.5604	5.28	-	0.27 ± 0.09	0.22 ± 0.05	7.17*****	0.0280
18:0/24:5	864.5714	864.5760	5.32	-	0.13 ± 0.03	0.26 ± 0.05	10.21*****	0.0127
**PI**	$[M-H]^-$	$[M-H]^-$ calc	diff (ppm)					
16:0/20:4	857.5152	857.5186	3.91	0.26 ± 0.11^{a}	0.37 ± 0.10	0.55 ± 0.09^{a}	7.89	0.0088

18:1e/20:4	869.5523	869.5549	3.03	0.47 ± 0.34	0.10 ± 0.04	0.15 ± 0.06	5.01	0.0311
17:0/20:4	871.5331	871.5342	1.26	0.13 ± 0.04[a]	0.34 ± 0.10[a,b]	0.24 ± 0.06[b]	11.23	0.0028
18:1/20:5	881.5154	881.5186	3.57	0.46 ± 0.27[a,b]	0.09 ± 0.01[a]	0.10 ± 0.02[b]	10.78	0.0032
18:1/20:4+ 18:0/20:5	883.5305	883.5342	4.19	3.01 ± 0.04[a,b]	1.08 ± 0.06[a,c]	1.05 ± 0.09[b,c]	721.85	>0.0001
18:0/20:4	885.5447	885.5499	5.81	3.68 ± 1.30	4.43 ± 0.20	5.68 ± 0.63	6.24	0.0174
19:1/20:4	897.5487	897.5499	1.28	0.13 ± 0.06[a]	0.24 ± 0.01[a,b]	0.14 ± 0.04[b]	13.00	0.0017
19:0/20:4	899.5624	899.5655	3.45	0.07 ± 0.04[a,b]	0.37 ± 0.07[a,c]	0.26 ± 0.06[b,c]	25.66	0.0001
20:1/20:5	909.5450	909.5499	5.33	1.69 ± 0.75[a,b]	0.48 ± 0.09[a]	0.45 ± 0.13[b]	14.10	0.0012
20:1/20:4	911.5604	911.5655	5.59	1.57 ± 0.14[a,b]	2.41 ± 0.06[a]	3.18 ± 0.41[b]	24.92	0.0001

*, **, *** Ref. Supplementary Table S1.

**** Predicted structures. Positions of alkyl/acyl groups in a glycerol backbone and double bound positions in alkyl chains were not determined.

***** $F_{1,8}$

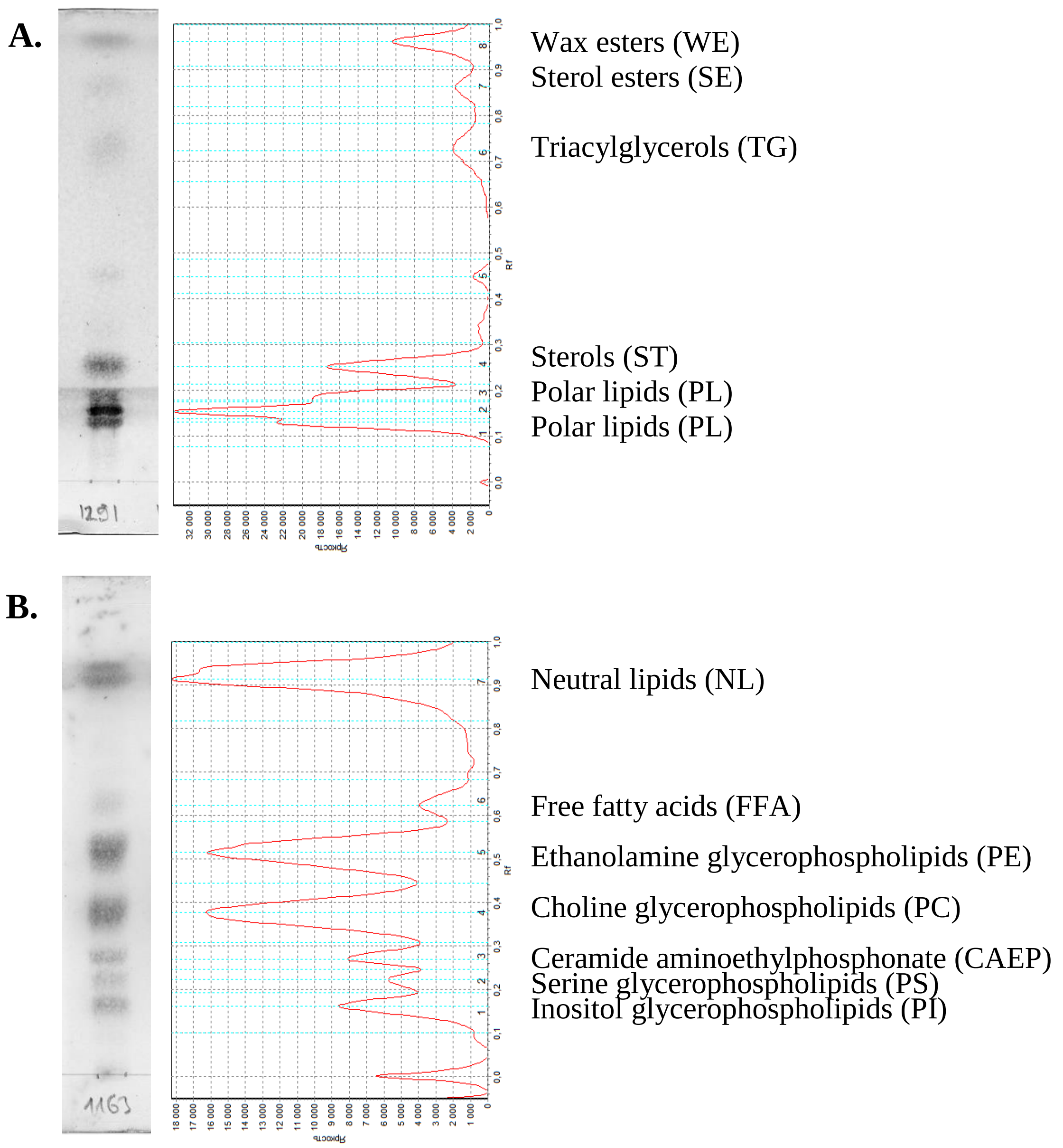

Supplementary Figure S1. Examples of TLC plates and densitometry analysis the composition of (**A**) total lipid classes and (**B**) polar lipid classes of the nudibranch molluscs from the Kuril Islands (the Sea of Okhotsk).

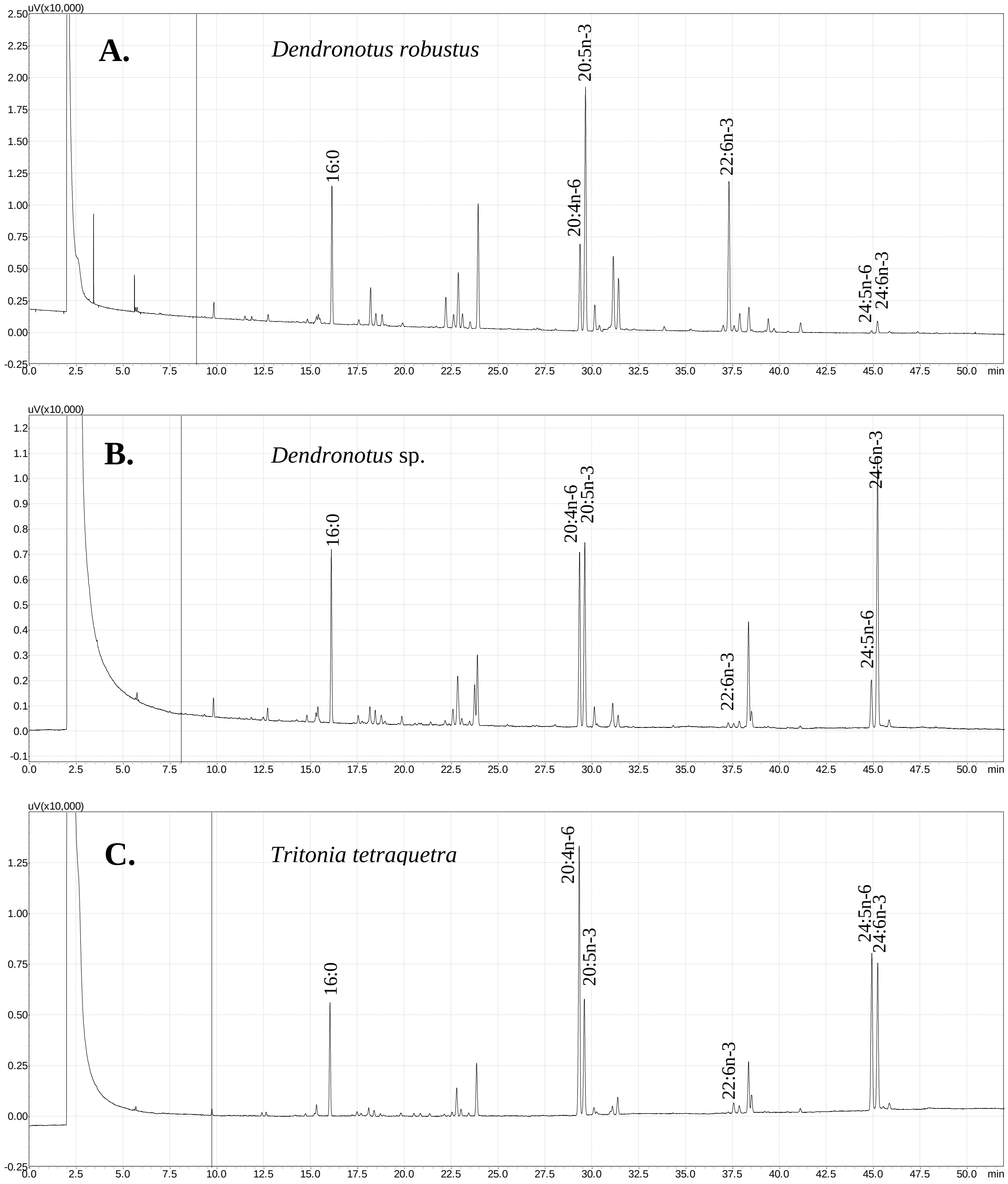

Supplementary Figure S2. Gas chromatography analysis of total fatty acid methyl esters obtained by hydrolysis of total lipids of the nudibranch molluscs (**A**) *Dendronotus robustus*, (**B**) *Dendronotus* sp., and (**C**) *Tritonia tetraquetra* from the Kuril Islands (the Sea of Okhotsk).

FAME 20:4n-6

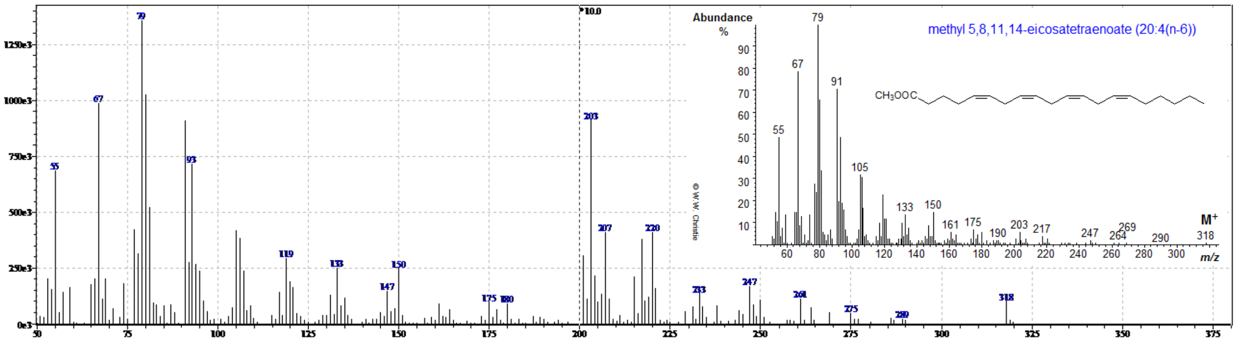

FAME 20:5n-3

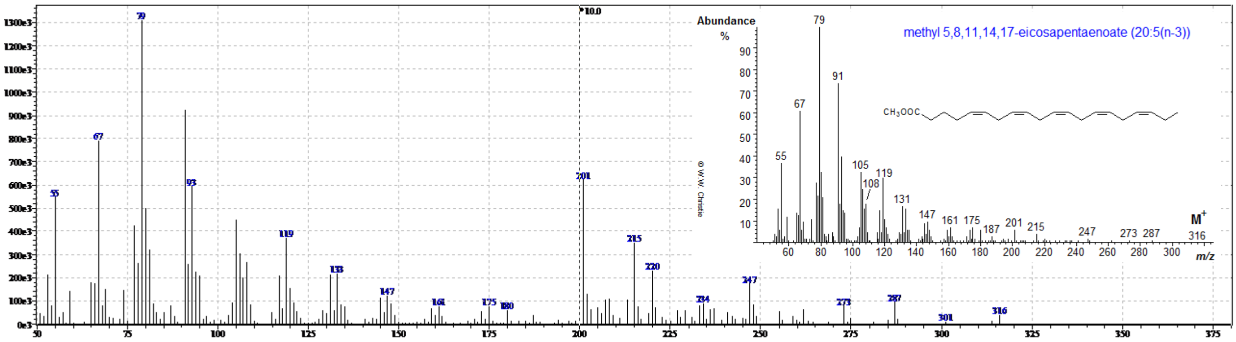

FAME 22:6n-3

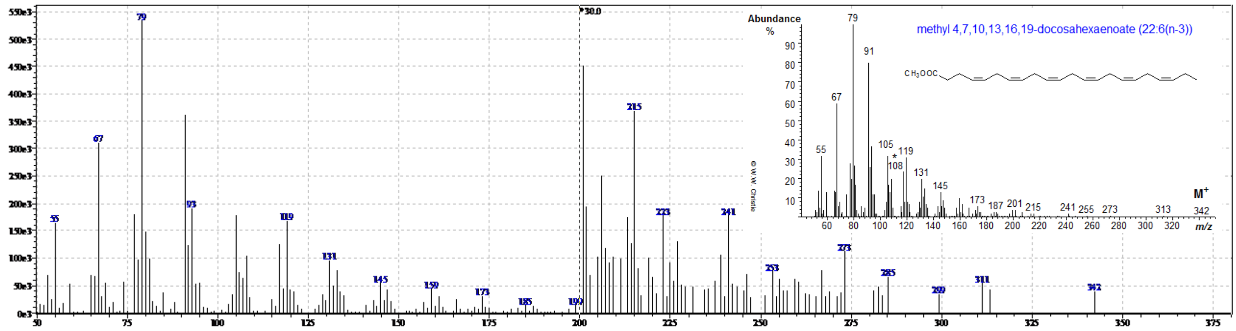

FAME 24:5n-6

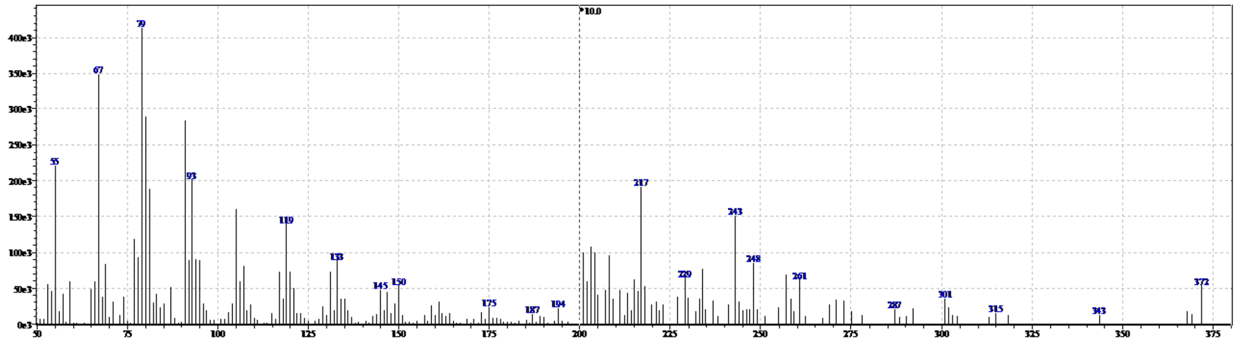

FAME 24:6n-3

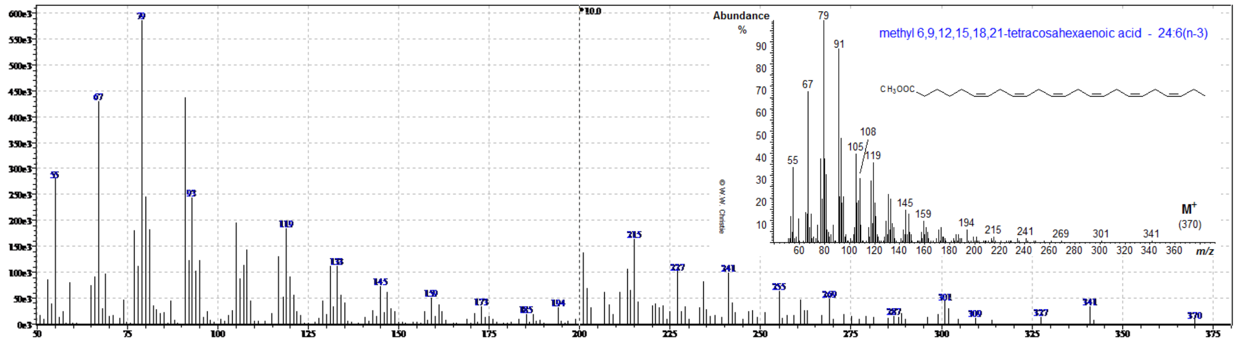

DMOX 20:4n-6

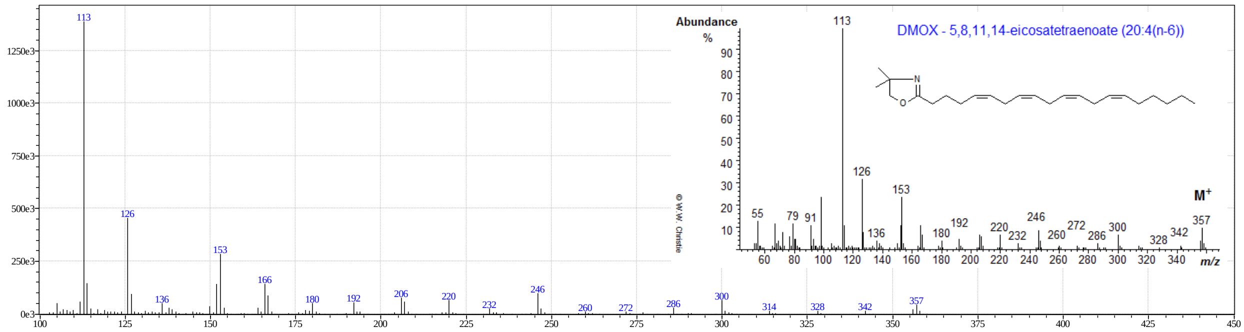

DMOX 20:5n-3

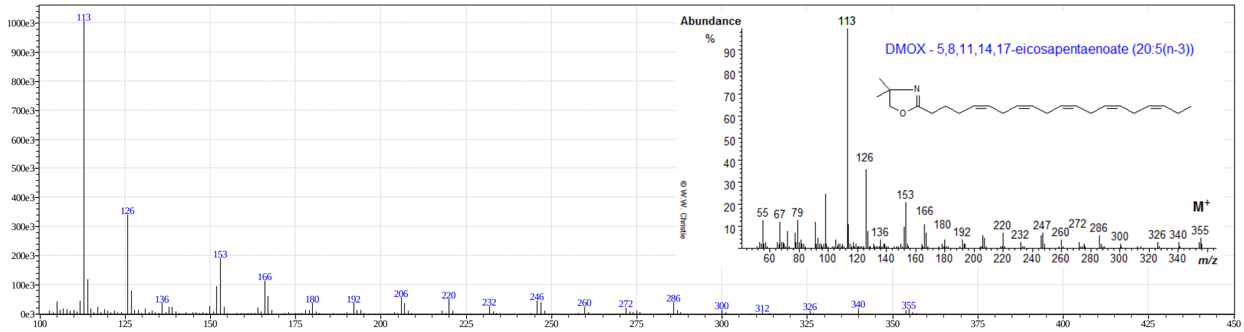

DMOX 22:6n-3

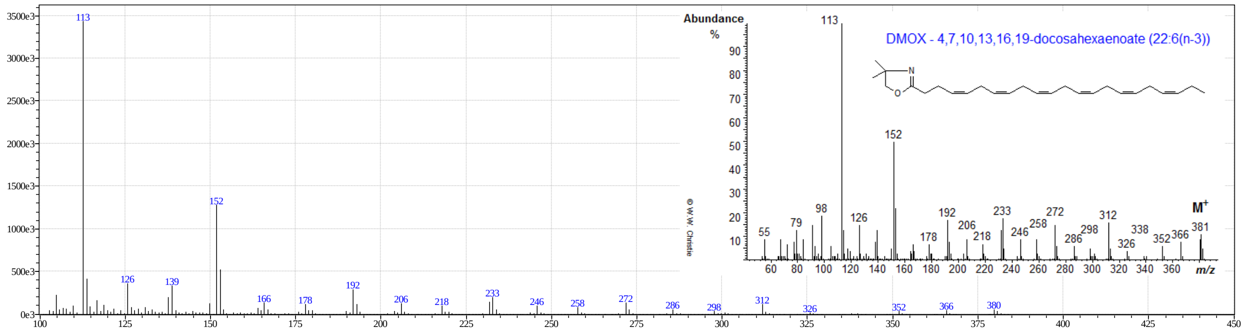

DMOX 24:5n-6

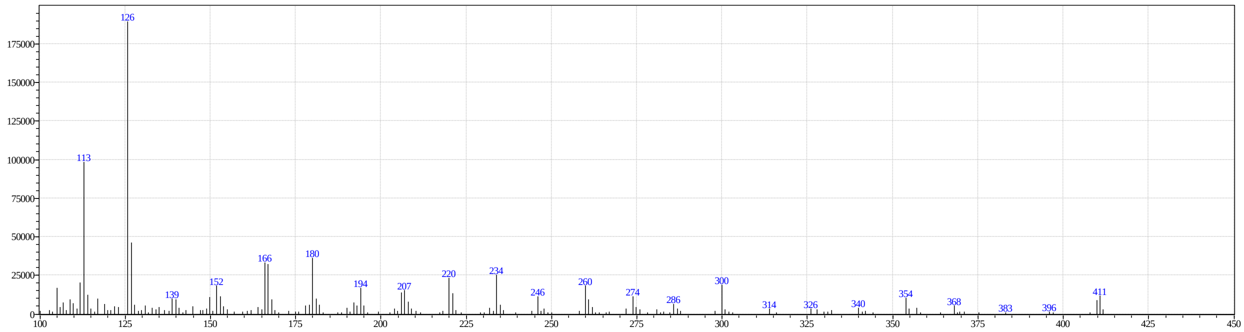

DMOX 24:6n-3

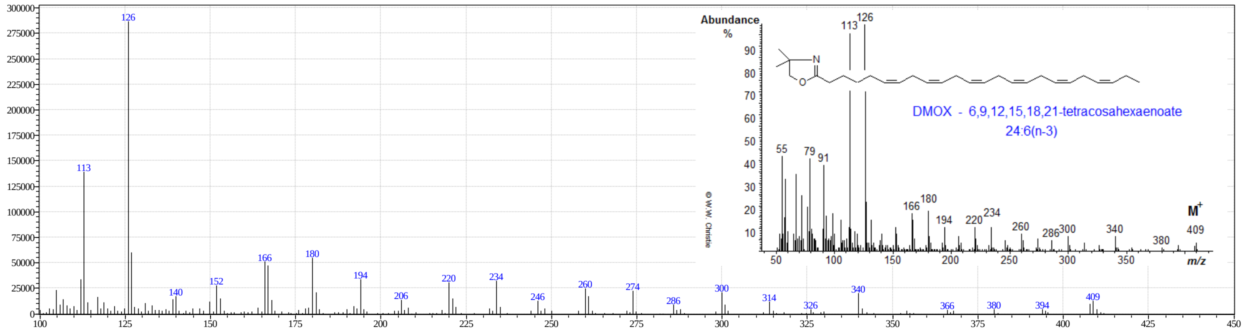

Supplementary Figure S3. Mass spectra of major polyunsaturated fatty acid methyl esters (FAME) and their 4,4-dimethyloxazoline derivatives (DMOX) obtained from total lipids of the nudibranch molluscs from the Kuril Islands (the Sea of Okhotsk). For reference, the correspondence spectrum from the Archive of mass spectra (Christie, W.W. The LipidWeb. Archive of mass spectra. (2019) https://www.lipidhome.co.uk/ms/methesters/me-arch/index.htm; https://www.lipidhome.co.uk/ms/dmox/dmox-arch/index.htm) were placed in the upper right corner of each spectrum obtained.

DMOX 22:2Δ7,13

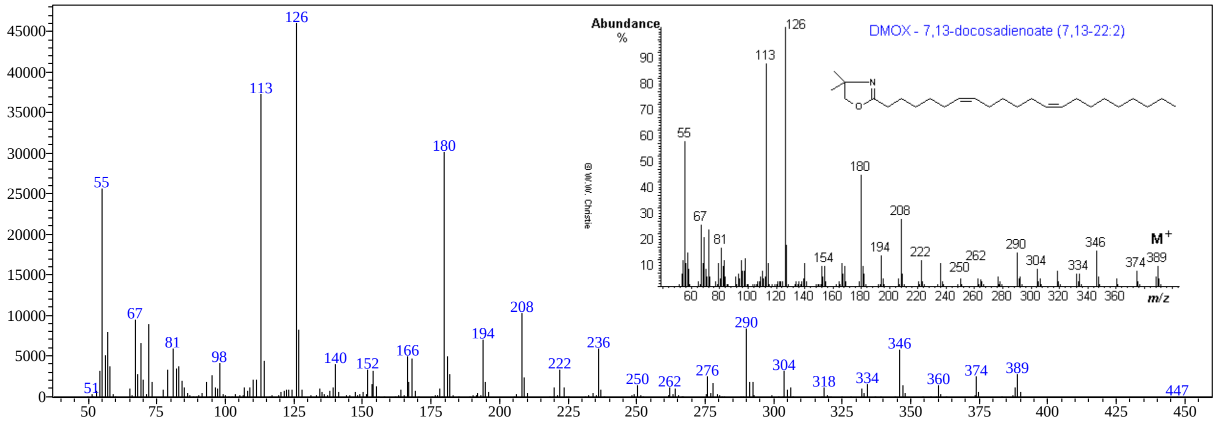

DMOX 22:2Δ7,15

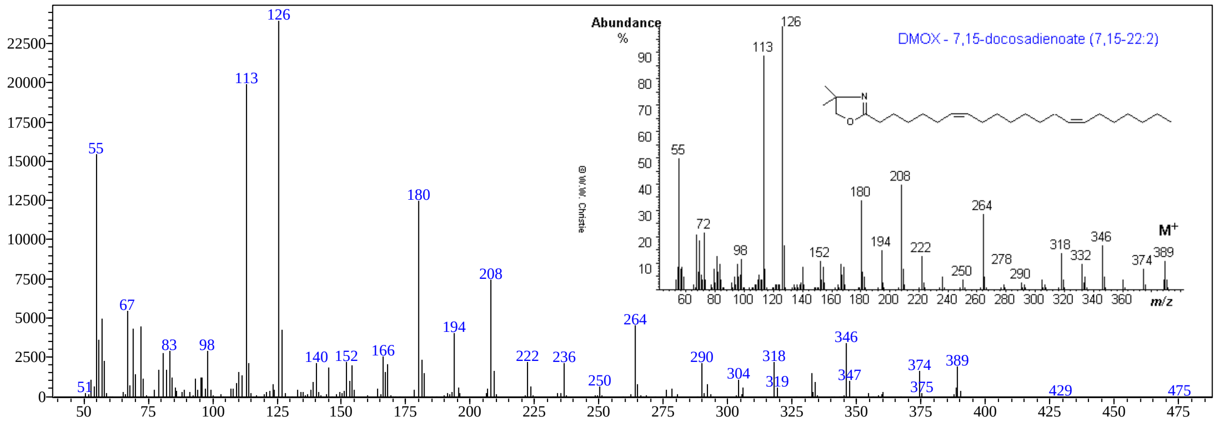

Supplementary Figure S4. Mass spectra 4,4-dimethyloxazoline derivatives (DMOX) of two non-methylene-interrupted (NMI) fatty acids obtained from total lipids of the nudibranch molluscs from the Kuril Islands (the Sea of Okhotsk). For reference, the correspondence spectrum from the Archive of mass spectra (Christie, W.W. The LipidWeb. Archive of mass spectra. (2019) https://www.lipidhome.co.uk/ms/methesters/me-arch/index.htm; https://www.lipidhome.co.uk/ms/dmox/dmox-arch/index.htm) were placed in the upper right corner of each spectrum obtained.

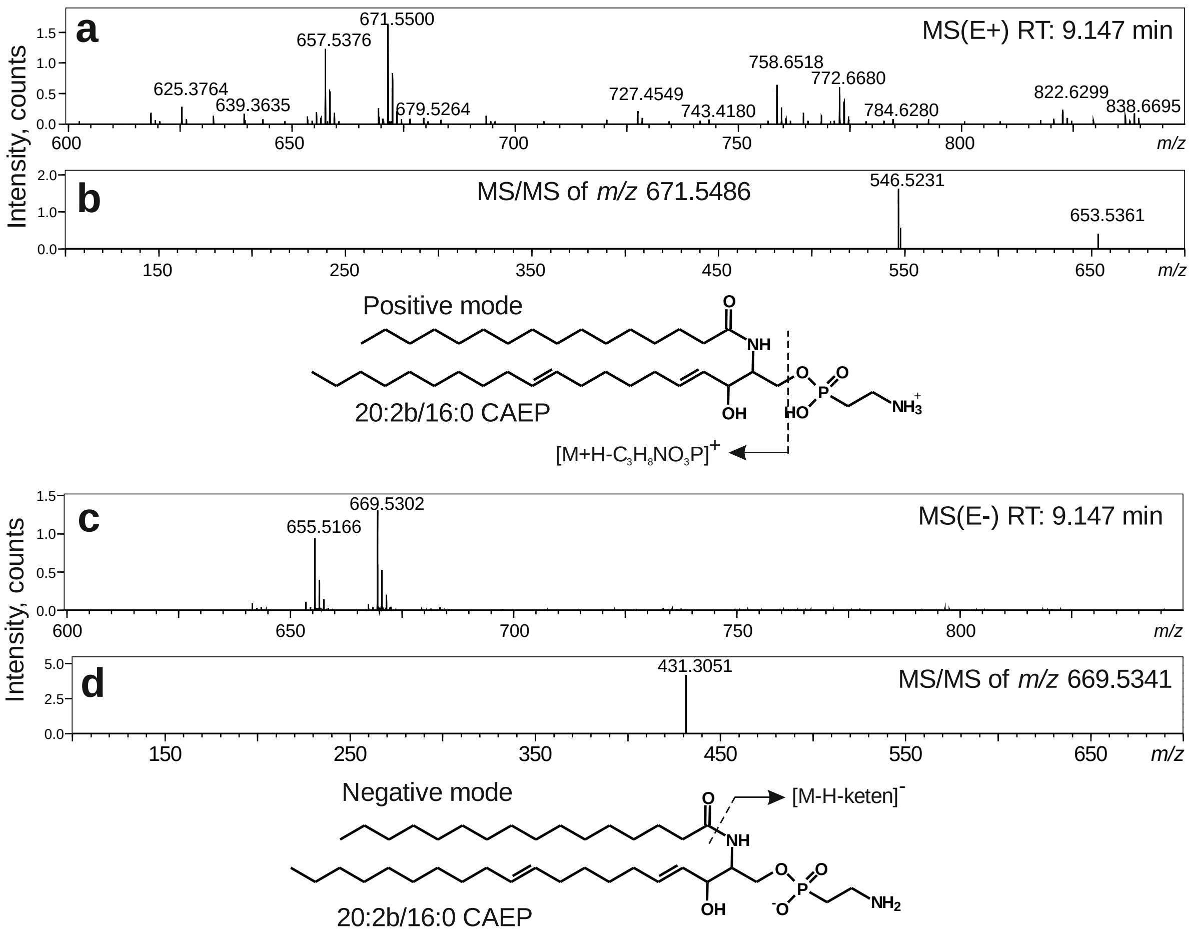

Supplementary Figure S5. Mass spectrometric fragmentation of ceramide aminoethylphosphonate (CAEP). Electrospray ionization mass spectra of CAEP molecule with a long-chain 20:2 sphingoid base and 16:0 *N*-acyl group, CAEP(20:2b/16:0). Total lipids of nudibranchs were analyzed by liquid chromatography – high resolution tandem mass spectrometry. Panel **a** shows the mass spectra for the peak eluting at 9.147 min, and panel **b** shows the MS/MS spectra of ions at *m/z* 671.5486 in positive (E+) mode. Panel **c** shows the mass spectra of the same peak, and panel **d** shows the MS/MS spectra of ions at *m/z* 669.5341 in negative (E-) mode. $[M+H]^+$ and $[M+H+Et_3N]^+$ ions, as well as $[M-H]^-$ ions, present in the mass spectrum of each CAEP molecular species. In the MS/MS spectrum (positive mode), the $[M+H]^+$ ion eliminated water (H_2O) or 2-aminoethylphosphonic acid ($C_2H_8NO_3P$) and formed two ions: $[M+H-H_2O]^+$ and $[M+H-C_2H_8NO_3P]^+$. In the MS/MS spectrum (negative mode), the $[M-H]^-$ ion lost the neutral keten and formed the main ion of aminoethylphosphonate of the long-chain base. These characteristic ions allowed us to determine the numbers of carbon atoms and double bonds both in long-chain bases and in *N*-acyl groups of each CAEP molecular species. The position of double bonds was not determined.

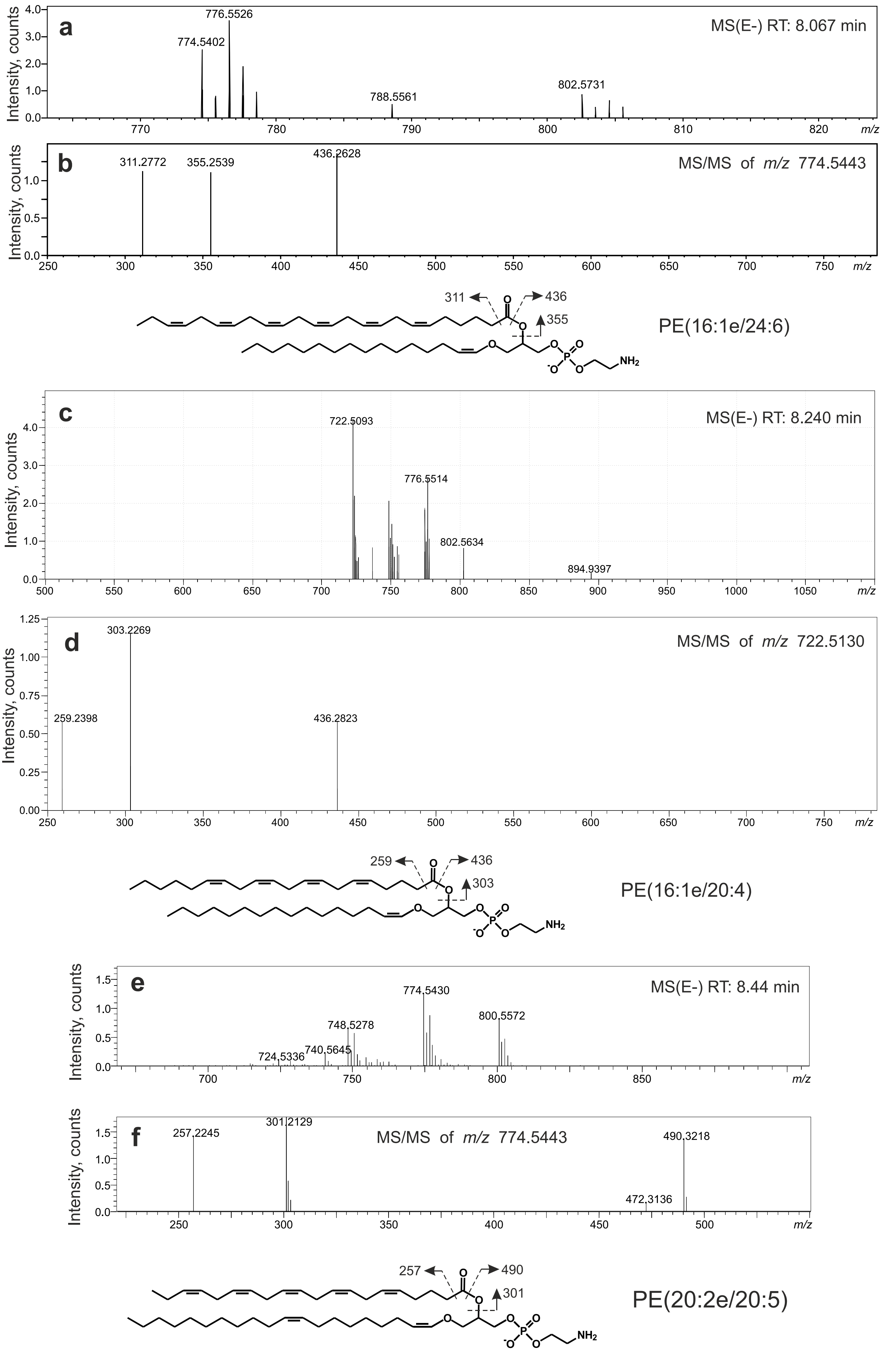

a
MS(E-) RT: 8.067 min
Intensity, counts
776.5526
774.5402
788.5561
802.5731
m/z
b
MS/MS of m/z 774.5443
311.2772
355.2539
436.2628
311
436
355
PE(16:1e/24:6)
NH2
c
MS(E-) RT: 8.240 min
722.5093
776.5514
802.5634
894.9397
d
MS/MS of m/z 722.5130
303.2269
259.2398
436.2823
259
436
303
PE(16:1e/20:4)
e
MS(E-) RT: 8.44 min
774.5430
748.5278
800.5572
724.5336
740.5645
f
MS/MS of m/z 774.5443
257.2245
301.2129
490.3218
472.3136
257
490
301
PE(20:2e/20:5)

Supplementary Figure S6. Electrospray ionization mass spectra of 1-*O*-hexadecenyl-2-tetracosahexaenoyl-*sn*-glycero-3-phosphoethanolamine PE(16:1e/24:6) and 1-*O*-hexadecenyl-2-arachidonoyl-*sn*-glycero-3-phosphoethanolamine PE(16:1e/20:4). The total lipids of nudibranchs were analyzed using normal phase liquid chromatography – high resolution tandem mass spectrometry (LC–HRMS) detecting negative ions. The mass spectra for the lipids eluting between 7.8-8.2 min (panel **a**), 8.0-8.4 min (panel **c**), and 7.6-9.4 (panels **e**) are shown. The MS/MS spectra of *m/z* 774.5443 (panel **b**), *m/z* 722.5130 (panel **d**), and *m/z* 774.5443 (panel **f**) are presented. The predicted structures of PE(16:1e/24:6), PE(16:1e/20:4), PE(20:2e/20:5), and the product ions are shown.

PE molecular species with RT = 8.07 min gave negative quasi-molecular ions $[M–H]^–$ at *m/z* 774.5402 corresponding to the composition $[C_{45}H_{78}NO_7P]^–$ (calculated 774.5443) (Fig. S6**a**). MS^2 fragmentation of these ions (Fig. S6**b**) resulted in the formation of anions at *m/z* 355.2539 ($[C_{24}H_{35}O_2]^–$) and *m/z* 311.2772 ($[C_{24}H_{35}O_2–CO_2]^–$), corresponding to 24:6 acid anions and decarboxylated 24:6 acid anions, respectively. The signal at *m/z* 436.2628 appeared in the MS^2 spectrum when the quasi-molecular ions $[M–H]^–$ lost a dehydrated molecule of 24:6 acid. According to the elemental composition calculated and the value of monoisotopic molecular mass, this component was identified as *O*-alkyl aclyl glycerophosphoethanolamine, PE(16:1e/24:6). Correspondingly, other major PE molecular species with $[M–H]^–$ ions at *m/z* 722.5093 ($[C_{41}H_{74}NO_7P]^–$, calculated 722.5130), which showed three ions at *m/z* 303.2269, 259.2398, and 436.2823 in the MS^2 spectrum, was identified as PE(16:1e/20:4) (Fig. S6**c**,**d**). Isobaric PE molecular species with $[M–H]^–$ ions at *m/z* 774.5402 ($[C_{45}H_{78}NO_7P]^–$, calculated 774.5443), which showed three ions at *m/z* 301.2129, 257.2245, and 490.3218 in the MS^2 spectrum, was identified as PE(20:2e/20:5) (Fig. S6**e**,**f**).

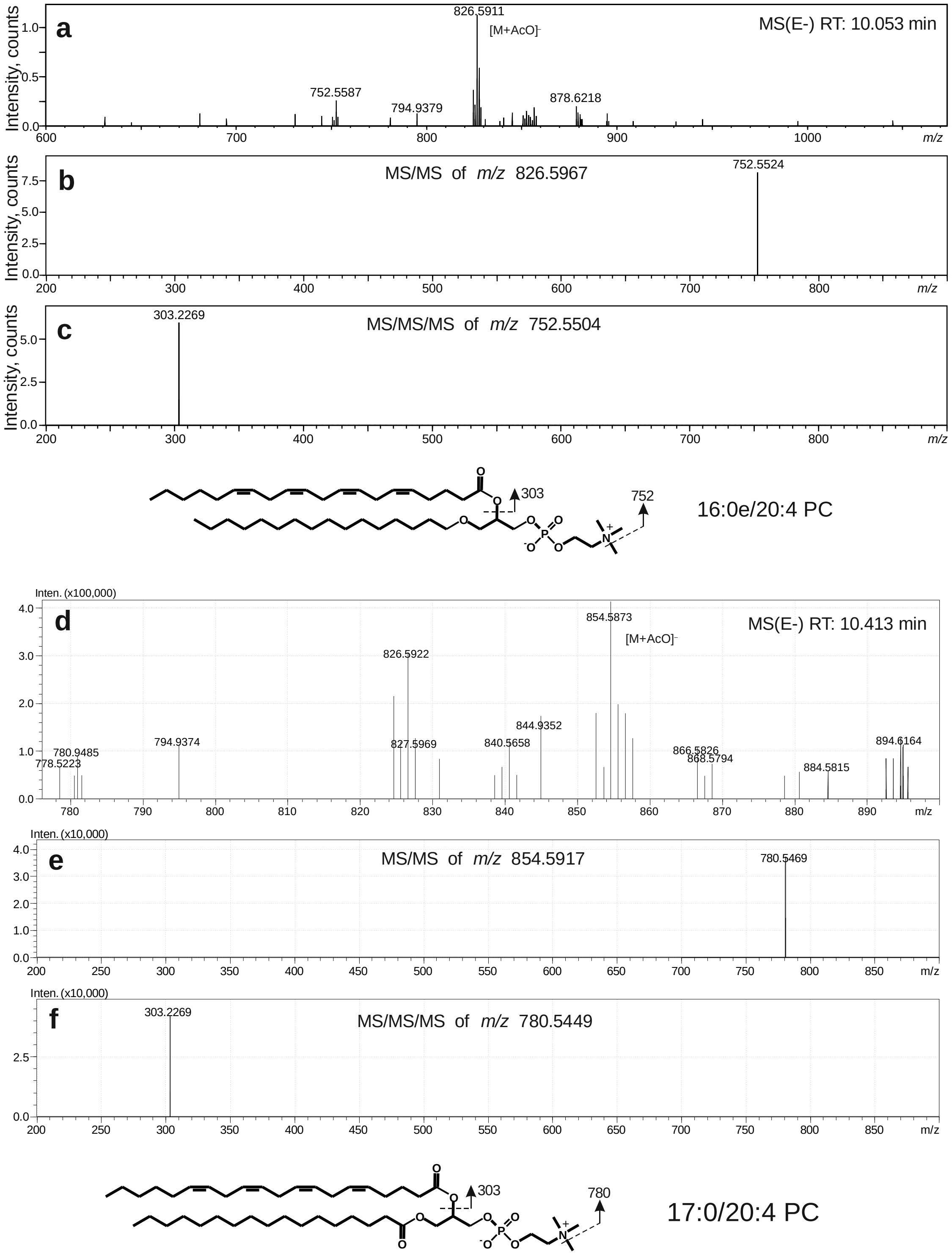

Supplementary Figure S7. Electrospray ionization mass spectra of 1-*O*-hexadecyl-2-arachidonoyl-*sn*-glycero-3-phosphocholine PC(16:0e/20:4) and 1-heptadecanoyl-2-arachidonoyl-*sn*-glycero-3-phosphocholine PC(17:0/20:4). The total lipids of the nudibranchs were analyzed using normal phase liquid chromatography – high resolution tandem mass spectrometry (LC–HRMS) detecting negative ions. Panels **a** and **d** show the mass spectra for the lipids (adducts with acetic acid) eluting between 9.8-10.2 and 10.2-10.6 min, respectively. The MS^2 spectrum of *m/z* 826.5967 and the MS^3 spectrum of *m/z* 752.5504 are shown in panels **b** and **c**, respectively. The

MS^2 spectrum of *m/z* 854.59177 and the MS^3 spectrum of *m/z* 780.5449 are shown in panels **e** and **f**, respectively. The predicted structures of the molecular species and the product ions are shown.

PC molecular species with RT = 10.05 min formed negative acetylated molecular ions $[M+CH_3COO]^-$ at *m/z* 826.5911 corresponding to the composition $[C_{44}H_{82}NO_7P+AcO]^-$ (calculated 826.5967) (Fig. S7**a**). At the MS^2 stage, these ions eliminated a molecule of $C_3H_6O_2$ (methyl acetate) and formed ions at *m/z* 752.5524 (Fig. S7**b**). At the MS^3 stage, the ions at *m/z* 752.5504 formed ions with *m/z* 303.2269 ($[C_{20}H_{31}O_2]^-$, calculated 303.2324) corresponding to 20:4 acid anions (Fig. S7**c**). On the base of the fragmentation pathways and the monoisotopic molecular mass values, this molecular species was identified as *O*-alkyl aclyl glycerophosphocholine, namely PC(16:0e/20:4). Generally, acetylated molecular ions of PE lost methyl acetate at the MS^2 stage, and then the subsequent fragmentation characterized acyl groups at the MS^3 stage. For example, negative acetylated molecular ions $[M+CH_3COO]^-$ at *m/z* 854.5873 corresponded to the composition $[C_{45}H_{82}NO_8P+AcO]^-$ (calculated 854.5917) and gave ions with *m/z* 780.55469 and 303.2269 at the MS^2 stage and the MS^3 stage, respectively (Fig. S7**d**,**e**,**f**). This molecular species with eight oxygen atoms was identified as diacyl glycerophosphocholine, PC (17:0/20:4).

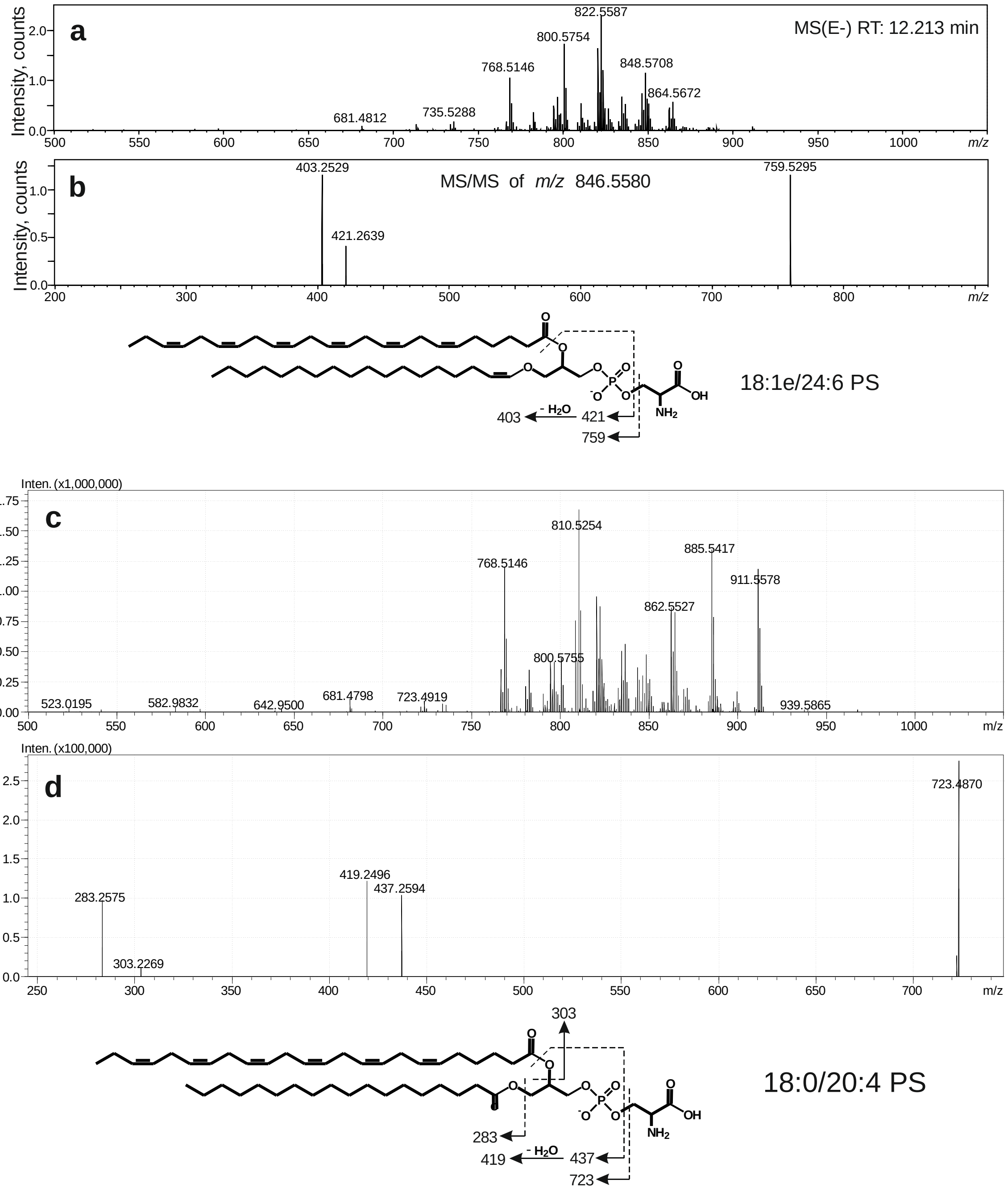

Supplementary Figure S8. Electrospray ionization mass spectra of 1-*O*-octadecenyl-2-tetracosahexaenoyl-*sn*-glycero-3-phosphoserine, PS(18:1e/24:6) and 1-octadecanoyl-2-arachidonoyl-*sn*-glycero-3-phosphoserine, PS(18:0/20:4). The total lipids of the nudibranchs were analyzed using normal phase liquid chromatography – high resolution tandem mass spectrometry (LC–HRMS) detecting negative ions. Panels **a** and **c** show the mass spectra for the lipids eluting between 12.1-12.4 minutes. The MS^2 spectra of *m/z* 846.5580 and *m/z* 810.5285 are shown in panels **b** and **d**, respectively. The predicted structures of the molecular species and the product ions are shown.

Among PS molecular species, the component with negative quasi-molecular ions $[M–H]^–$ at *m/z* 846.5580 was detected (Fig. S8**a**). The elemental composition of the ions was calculated as $[C_{48}H_{82}NO_9P]^–$. The MS^2 spectrum of $[M–H]^–$ (Fig. S8**b**) contained a signal of characteristic ion $[M–H–C_3H_5NO_2]^–$ at *m/z* 759.5295 corresponding to the loss of serine group with monoisotopic mass of 87.0359 ($C_3H_5NO_2$, calculated 87.0320). The MS^2 spectrum also contained a signal at *m/z* 421.2639 corresponding to the quasi-molecular ion, which lost simultaneously serine and 24:6 acyl group. Carboxylate anion signals were not observed. This fragment is characteristic for alkyl acyl and alkenyl acyl glycerophosphoserines. Therefore, this molecular species was identified as PS(18:1e/24:6). The MS^2 spectra of $[M–H]^–$ of diacyl PS(18:0/20:4) also contained signals of characteristic ions corresponding to the loss of serine group and the loss of serine and acyl groups, but carboxylate anion signals were observed (Fig. S8**c**,**d**).

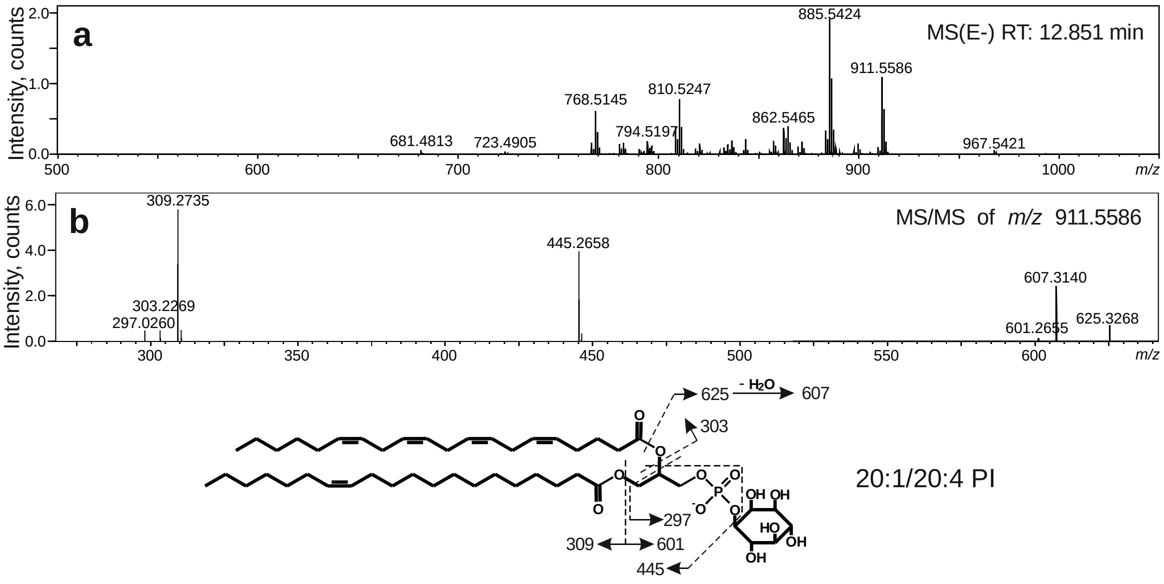

Supplementary Figure S9. Electrospray ionization mass spectra of 1-eicosenoyl-2-arachidonoyl-*sn*-glycero-3-phosphoinositol (20:1/20:4 PI). The total lipids of the nudibranchs were analyzed using normal phase liquid chromatography – high resolution tandem mass spectrometry (LC–HRMS) detecting negative ions. Panel **a** shows the mass spectrum for the lipids eluting between 12.7 and 13.0 minutes. The MS/MS spectrum of *m/z* 911.5586 is shown in panel **b**. The predicted structures of the molecular species and the product ions are shown below panel **b**.

PI molecular species with RT = 12.851 min produced negative quasi-molecular ions $[M–H]^–$ at *m/z* 911.5586 corresponding to $[C_{49}H_{85}O_{13}P]^–$ (Fig. S9**a**). MS^2 fragmentation of the ions $[M–H]^–$ gave several characteristic ions (Fig. S9**b**). The ions at *m/z* 303.2269 $[C_{20}H_{31}O_2]^–$ and 309.2735 $[C_{20}H_{37}O_2]^–$ corresponded to carboxylate anions of 20:4 and 20:1, respectively. The loss of 20:4 and 20:1 gave ions at *m/z* 607.3140 ($[M–H–C_{20}H_{32}O_2]^–$) and 601.2655 ($[M–H–C_{20}H_{38}O_2]^–$), respectively. The loss of dehydrated 20:4 led to the formation of ions at *m/z* 625.3268. The appearance of ions at *m/z* 297.0260 (calculated 297.0381) was caused by the simultaneous loss of 20:4 and 20:1. The loss of inositol and 20:4 acyl fragments $[M–H–(C_6H_{10}O_5 + C_{20}H_{32}O_2)]^–$ gave the ions at *m/z* 445.2658, characteristic for PI. Thus, the molecular species was identified as PI (20:1/20:4).

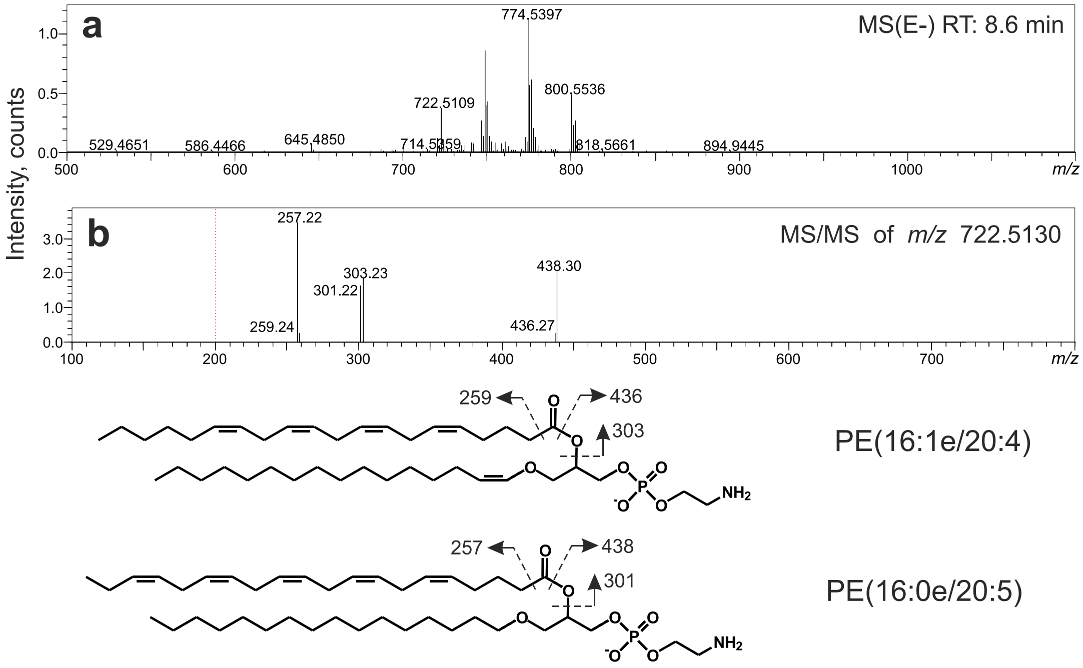

Supplementary Figure S10. Electrospray ionization mass spectra of a mixture of isobaric 1-*O*-hexadecenyl-2-arachidonoyl-*sn*-glycero-3-phosphoethanolamine PE(16:1e/20:4) and 1-*O*-hexadecyl-2-eicosapentaenoyl-*sn*-glycero-3-phosphoethanolamine PE(16:1e/20:4). The total lipids of nudibranchs were analyzed using normal phase liquid chromatography – high resolution tandem mass spectrometry (LC–HRMS) detecting negative ions. Panel **a** shows the mass spectrum for the lipids eluting between 8.43-9.11 min. Panel **b** shows the MS/MS spectrum of ions at *m/z* 722.5109 ($[C_{41}H_{74}NO_7P]^-$, calculated 722.5130). The predicted structures of PE(16:1e/20:4), PE(16:0e/20:5) and the product ions are shown.
